# Looking for a Better Characterization of Triple-Negative Breast Cancer by Means of Circulating Tumor Cells

**DOI:** 10.3390/jcm9020353

**Published:** 2020-01-27

**Authors:** Manuel Abreu, Pablo Cabezas-Sainz, Thais Pereira-Veiga, Catalina Falo, Alicia Abalo, Idoia Morilla, Teresa Curiel, Juan Cueva, Carmela Rodríguez, Vanesa Varela-Pose, Ramón Lago-Lestón, Patricia Mondelo, Patricia Palacios, Gema Moreno-Bueno, Amparo Cano, Tomás García-Caballero, Miquel Ángel Pujana, Laura Sánchez-Piñón, Clotilde Costa, Rafael López, Laura Muinelo-Romay

**Affiliations:** 1 Liquid Biopsy Analysis Unit, Translational Medical Oncology (Oncomet), Health Research Institute of Santiago (IDIS), 15706 Santiago de Compostela, Spain; maabreu20@gmail.com (M.A.); alicia.abalo.pineiro@sergas.es (A.A.); Ramon.Manuel.Lago.Leston@sergas.es (R.L.-L.); patricia_mondelo@hotmail.com (P.M.); rafael.lopez.lopez@sergas.es (R.L.); 2 Department of Zoology, Genetics and Physical Anthropology, Universidade de Santiago de Compostela, Campus de Lugo, 27002 Lugo, Spain; pablo.cabezas@rai.usc.es (P.C.-S.); lauraelena.sanchez@usc.es (L.S.-P.); 3 Roche-CHUS Joint Unit, Oncomet, Health Research Institute of Santiago (IDIS), Complejo Hospitalario de Santiago de Compostela, Trav. Choupana s/n, 15706 Santiago de Compostela, Spain; thaispv85@gmail.com (T.P.-V.); clotildecnogueira@gmail.com (C.C.); 4 Department of Medical Oncology-Breast Cancer Unit, Institut Català d’Oncologia (ICO)-Hospitalet-Institut d’Investigació Biomèdica de Bellvitge (IDIBELL), Universitat de Barcelona, 08007 Barcelona, Spain; cfalo@iconcologia.net (C.F.); idoiamorilla@gmail.com (I.M.); 5 Translational Medical Oncology (Oncomet), Health Research Institute of Santiago de Compostela (IDIS), Complexo Hospitalario Universitario de Santiago de Compostela (SERGAS), 15706 Santiago de Compostela, Spain; mtcuriel@hmhospitales.com (T.C.); jfcueva@gmail.com (J.C.); rodriguezlopez.carmela@gmail.com (C.R.); vanesa.var.p@gmail.com (V.V.-P.); ppalaciosozores@yahoo.es (P.P.); 6 Centro de Investigación Biomédica en Red de Cáncer (CIBERONC), Monforte de Lemos 3-5, 28029 Madrid, Spain; gmoreno@iib.uam.es (G.M.-B.); acano@iib.uam.es (A.C.); 7 Fundación MD Anderson Internacional, C/Gómez Hemans 2, 28033 Madrid, Spain; 8 Departamento de Bioquímica, Universidad Autónoma de Madrid (UAM), Instituto de Investigaciones Biomédicas ‘Alberto Sols’ (CSIC-UAM), IdiPaz, Arzobispo Morcillo 4, 28029 Madrid, Spain; 9 Departamento de Ciencias Morfológicas, Facultad de Medicina, Universidad de Santiago, Servicio de Anatomía Patológica, Complejo Hospitalario Universitario de Santiago, 15706 Santiago de Compostela, Spain; tomas.garcia-caballero@usc.es; 10 ProCURE, Catalan Institute of Oncology (ICO), Bellvitge Institute of Biomedical Research (IDIBELL), 08908 Barcelona, Spain; miguelangel.pujana@gmail.com

**Keywords:** triple-negative breast cancer (TNBC), circulating tumor cells (CTCs), metastasis, cell plasticity, epithelial to mesenchymal transition, stemness, tumor biomarkers, tissue inhibitor of metalloproteinases 1, androgen receptor, therapeutic targets

## Abstract

Traditionally, studies to address the characterization of mechanisms promoting tumor aggressiveness and progression have been focused only on primary tumor analyses, which could provide relevant information but have limitations to really characterize the more aggressive tumor population. To overcome these limitations, circulating tumor cells (CTCs) represent a noninvasive and valuable tool for real-time profiling of disseminated tumor cells. Therefore, the aim of the present study was to explore the value of CTC enumeration and characterization to identify markers associated with the outcome and the aggressiveness of triple-negative breast cancer (TNBC). For that aim, the CTC population from 32 patients diagnosed with TNBC was isolated and characterized. This population showed important cell plasticity in terms of expression of epithelia/mesenchymal and stemness markers, suggesting the relevance of epithelial to mesenchymal transition (EMT) intermediate phenotypes for efficient tumor dissemination. Importantly, the CTC signature demonstrated prognostic value to predict the patients’ outcome and pointed to a relevant role of tissue inhibitor of metalloproteinases 1 (TIMP1) and androgen receptor (AR) for TNBC biology. Furthermore, we also analyzed the usefulness of the AR and TIMP1 blockade to target TNBC proliferation and dissemination using in vitro and in vivo zebra fish and mouse models. Overall, the molecular characterization of CTCs from advanced TNBC patients identifies highly specific biomarkers with potential applicability as noninvasive prognostic markers and reinforced the value of TIMP1 and AR as potential therapeutic targets to tackle the most aggressive breast cancer.

## 1. Introduction

Breast cancer (BC) is the leading cause of cancer-related deaths in women despite prevention, research and advances in treatment [1]. There are different subtypes of BC that are usually classified according to the status of the estrogen (ER) and progesterone (PR) receptors and the human epidermal growth factor receptor 2 (HER2). During the last years, the use of targeted therapies in BC patients has increased the treatment benefit and improved prognosis and patient survival for ER^+^ and HER2^+^ tumors. However, the triple-negative breast cancer (TNBC) subtype, characterized by the lack of ER and PR expression and no overexpression of HER2, remains a clinical challenge, because of the lack of specific therapeutic targets and the aggressive evolution of the disease [2].

Traditionally, characterization of the mechanisms behind tumor aggressiveness and progression have been mainly based on primary tumor samples, which could provide relevant information for specific tumor types but that may underscore the molecular complexity of the most aggressive tumor cells that can be diluted within the whole tumor. The characterization of circulating tumor cells (CTCs) constitutes a valuable alternative tool in the identification of new biomarkers and therapeutic targets to improve the management of cancer patients, since they provide a comprehensive picture of the disease, because they come from different tumor locations present in the patients [3].

Several studies have evidenced the presence of CTCs in TNBC patients at different stages, although the percentage is higher in metastatic patients. More importantly, CTC detection after surgery or during the adjuvant therapy administration has been associated with poor survival rates in these patients [3,4,5,6,7]. Besides, in accordance with the unfavorable prognosis of these tumors, CTC clusters are more often found in TNBC patients, indicating the existence of specially active dissemination mechanisms in these patients [8].

In addition to the prognostic value of the CTC monitoring in patients with TNBC, recent work has focused on their molecular characterization. Thus, Agelaki et al., characterized the CKs, ER, PR, EGFR and HER2 status on CTCs from a cohort of 10 metastatic TNBC- and 21 hormone receptor (HR)-positive patients, with 40% of the CTCs isolated being positive for CKs and EGFR, and the patients with CTCs negative for hormone receptor being those with worst survival rates [9]. Importantly, there are data evidencing that CTC characteristics change during tumor cell dissemination, favoring the intravasation, their survival in circulation and their final colonization. These changes are mainly related to the process of epithelial to mesenchymal transition (EMT) [10,11]. This process is associated with a reduced expression of epithelial markers, along with increased plasticity and aggressiveness, making these cells more resistant to cell death and senescence [12]. In fact, it is well known that EMT and stemness markers expression in this circulating tumor population could facilitate the resistance to chemotherapy and promote their capacity to metastasize [13,14,15,16,17].

Taking into account this knowledge and the need for new strategies to improve the alternatives for patients with TNBC, the present work aims to further study CTCs from these patients to identify new biomarkers and therapeutic targets. To that end, CTCs were immunoisolated from a cohort of 32 patients with stage III and IV TNBC and characterized using a panel of genes related to cancer aggressiveness and cell plasticity. The expression signature identified in CTCs from these patients was associated with a hybrid EMT status and a stem-like phenotype. Notably, from the CTC gene expression signature, AR and TIMP1 were studied as potential targets to block tumor growth and dissemination in TNBC.

## 2. Experimental Section

### 2.1. Patient Inclusion and Sample Collection

A total of 32 patients diagnosed with TNBC at Complexo Hospitalario Universitario de Santiago de Compostela, MD Anderson Cancer Center and Institut Catalá d’Oncologia (ICO), were included in the study (Table 1) from 2014 to 2017. In addition, 30 aged-matched healthy people (mean age 56.1, range 42–78 years), with an absence of a previous cancer episode, were also included as controls at Complexo Hospitalario Universitario de Santiago de Compostela. This study was approved by the Galician Ethical Committee (code 2013/462) and all samples were collected after signing the pertinent informed consent.

Tissue samples from patients included in this study were provided by the BioBank Complejo Hospitalario Universitario de Santiago (CHUS) (PT17/0015/0002), integrated in the Spanish National Biobanks Network; they were processed following standard operating procedures with the appropriate approval of the Ethical and Scientific Committees. These samples were composed of healthy tissue and tumor tissue from the primary tumor and/or metastasis when they were available. For the tumor tissues, at least the 80% of the sample was required to be composed of tumor cells. Microdissection was employed in some samples to reach this ratio.

Two tubes (7.5 mL) of peripheral blood were obtained from each patient: one EDTA vacutainer (Becton Dickinson) for CTC enrichment and characterization by CELLection^TM^ Epithelial Enrich Kit (Life Technologies, Ås Municipality, Norway), and one CellSave Preservative tube (Menarini, Silicon Biosystems Inc., Huntington Valley, PA, USA) for CTCs enumeration using the CellSearch System (Menarini, Silicon Biosystems Inc., Huntington Valley, PA, USA).

### 2.2. CTC Immunoisolation

A total of 7.5 mL of blood was employed for CTCs enumeration by the CellSearch System, using CellSearch Epithelial Circulating Tumor Cell Kit (Menarini, Silicon Biosystems Inc, Huntington Valley, PA, USA). This system automatically immunoisolated EpCAM^+^ CTCs, incubating the blood with ferrofluids coated with an anti-EpCAM antibody (clone VU1D9). Before this incubation the 7.5 mL of blood were centrifuged at 600 × *g* for 10 min at RT. The system removed the plasma fraction and incubated the cell fraction with the ferrofluids. After the isolation using a magnetic field, the system labeled the enriched cells with phycoerythrin (PE) conjugated anti-cytokeratins (CKs) antibodies, with allophycocyanin (APC) conjugated anti-CD45 antibodies and with 4,6-diamino-2-phenylindole (DAPI) to identify the nucleus. The CellTracks Analyzer (Menarini, Silicon Biosystems Inc, Huntington Valley, PA, USA) was then used to acquire digital images of the three different fluorescent dyes using a 12-bit camera; these images were reviewed by trained operators in order to determine the CTC count. Only round/oval, intact DAPI^+^_,_ CK^+^_,_ CD45^-^ cells were considered as CTCs.

The other 7.5 mL of blood (collected in EDTA tube) was used for isolation of EpCAM^+^ CTCs using CELLection^TM^ Epithelial Enrich Kit (Life Technologies, Ås Municipality, Norway) according to manufacturer’s instructions. The whole blood sample was centrifuged at 600 × *g* for 10 min at room temperature. Plasma was removed and the cell fraction was incubated with dynabeads coated with the anti-EpCAM antibody (clone Ber-EP4) and isolated, generating a magnetic field. After the enrichment step, CTCs coupled to the magnetic beads were resuspended in 100 μL of RNAlater (Ambion/Life Technology, Carlsbad, CA, USA) and stored at −80 °C until RNA extraction.

### 2.3. Gene Expression Analysis by RT-qPCR

The CTCs fraction obtained after isolation of EpCAM^+^ CTCs using CELLection^TM^ Epithelial Enrich Kit (Life Technologies, AS, Norway) was characterized as previously was described by Barbazán et al. [18]. RNA was purified with the QIAmp viral RNA mini kit (Qiagen, Hilden, Germany), specifically designed for very low cellularity samples. The cDNA was synthesized by using SuperScriptIII chemistry (Invitrogen, Alameda, CA, USA) following manufacturer’s instructions. To further optimize the sensibility of detection, we performed a preamplification step by using the TaqMan PreAmp Master Mix kit (Applied Biosystems, Foster City, CA, USA) with 14 reaction cycles. Preamplified products were subjected to TaqMan real-time PCR amplification for candidate genes (*CDH1*, *EPCAM*, *VIM*, *ZEB1*, *ZEB2*, *LOXL2*, *SNAI1*, *ANXA2*, *TIMP1*, *CRIPTO1*, *AR*, *ALDH1*, *ALDH2*, *CD133*, *CD49F*, *CD44*, *BCL11A* and *GAPDH*) using a 7500 real-time PCR System (Applied Biosystems, Foster City, CA, USA) and specific TaqMan assays (Table S1). Expression values for each gene were normalized to *CD45*, as a marker of nonspecific isolation of blood cells.

RNA was extracted from all FFPE samples (three cuts of 10 μm) with the miRNeasy FFPE Extraction Kit (Qiagen, Hilden, Germany) following the manufacturer’s instructions and using a solution for deparaffinization (Qiagen, Hilden, Germany). RNA from cell lines was obtained using High Pure RNA Isolation Kit (Roche Applied Science, Indianapolis, IN, USA) following the manufacturer’s instructions. Twenty ng of total RNA obtained from tissue samples and cell lines were reverse-transcribed using Amp ARN (Applied Biosystems, Foster City, CA, USA). Gene expression was then assessed using a 7500 real-time PCR System (Applied Biosystems, Foster City, CA, USA) and specific TaqMan assays (Table S1). Expression values for each gene were normalized to *GAPDH* as a reference gene.

### 2.4. Cell Lines

MDA-MB-231 cell line was acquired from the ATCC. The cells were authenticated by STR-profiling according to ATCC guidelines and cultured at 37 °C in a humid atmosphere with 5% CO_2_ and cultured in McCoy’s 5A medium (Gibco, Grand Island, NY, USA) supplemented with 10% fetal bovine serum (FBS, Gibco, South America) and 1% penicillin-streptomycin (Gibco, Grand Island, NY, USA).

### 2.5. TIMP1 Knock-Down

With the goal to decrease the expression of *TIMP1* in the MDA-MB-231cell line, lentiviral particles containing commercial constructs were used to block the translation of the mRNA that gives rise to the protein (Mission Lentiviral Transduction Particles, Sigma, St. Louis, MO, USA). Four different shRNAs were used, following the manufacturer’s instructions, employing a multiplicity of infection (MOI) of 10 and a Polybrene (Hexadimethylbromide; Sigma) final concentration of 8 μg/mL. Commercial particles containing a shRNA directed against a sequence not present in mammals (Mission Non-Mammalian shRNA Control Transduction Particles, Sigma, St. Louis, MO, USA) were used as control. The silenced lines were selected in the presence of puromycin (5 μg/mL) and named as SH2 and SH4 while the control was named as PLKO. The efficacy of the silencing was confirmed by RT-q-PCR and Western blot.

### 2.6. Western Blot

TIMP1, PI3K and AKT status was assessed by Western blot in 10% acrylamide gels, using anti-TIMP1 antibody 1:150 (AF970; R&D Systems, MN, USA), anti-AKT antibody 1:1000 (40D4, Cell Signaling, MA, USA), anti-pAKT 1:1000 (4060S, Cell Signaling, MA, USA), anti-PI3K 1:1000 (4292, Cell Signaling, MA, USA) and anti-pPI3K 1:1000 (4228S, Cell Signaling, MA, USA) to incubate the blots overnight at 4 °C. Afterward, the blots were incubated with a horseradish peroxidase-conjugated goat anti-rabbit polyclonal secondary antibody (1:5000; SC2354; Santa Cruz Biotechnology, CA, USA) or peroxidase-conjugated goat anti-mouse polyclonal secondary antibody (1:1000; SC2005; Santa Cruz Biotechnology, CA, USA) for 1 h at room temperature. Signals were detected with an enhanced chemiluminescence kit (Thermo Fisher, Waltham, MA, USA) following the manufacturer’s instructions; β-actin was used as housekeeping control.

### 2.7. Transwell Migration Assay

Migration assays were carried out using transwells with polycarbonate membrane, with a pore size of 8.0 μm (Corning, NY, USA), in 24-well plates; 5 × 10 ^4^ cells were seeded in transwells contained in 100 μL of serum-free culture medium. The wells of the plate were filled with 500 μL of complete culture medium with 20% FBS as chemoattractant. After 24 h of incubation, transwells were placed in a well with 500 μL of trypsin to detach the cells that had passed through the membrane. These cells were labeled using Calcein Acetomethyl Ester (4 μM, Invitrogen, Carlsbad, CA, USA), according to the manufacturer’s instructions. The fluorescence emitted by the cells was measured at 485 nm using a FLUOstar Optima fluorometer (BMG labtech, DE) in 96-well plates. For Abiraterone treatment, cells were incubated during the assay with 10 μM of Abiraterone, with DMSO 1% being the control condition.

### 2.8. Proliferation Assay

Proliferation assays were performed in 96-well plates, where 10^4^ cells were seeded per well in 200 μL of complete culture medium (10% FBS). Once the cells adhered to the plate (4 h), time 0 was measured. For this, the culture medium was removed and more medium was added with Alamar Blue (Invitrogen, Carlsbad, CA, USA) in a 1:10 dilution. Cells were then incubated for 3 h at 37 °C and the fluorescence was measured using a FLUOstar Optima fluorometer (BMG labtech, Ortenberg, Germany) at 544 nm. Subsequently, another plate was measured at 72 h, in order to establish the proliferation ratio. For androgen pathway inhibition, cells were incubated during the 72 h with 10 μM of Abiraterone, with DMSO 1% being the control condition.

### 2.9. Colony Formation Assay

In order to evaluate the colony formation capacity of the cells, agarose assays (Promega, Madison, WI, USA) were carried out in 96-well agar coated plates. A measurement was made at time 0 and another measurement at 96 h, being able to determine the colony formation occurred during this time interval. The measurement was made using the Alamar Blue method (Invitrogen, Carlsbad, CA, USA). This reagent was added in each well to a final concentration of 10%. After 3 h of incubation at 37 °C the measurement was carried out with a fluorometer at 544 nm. For Abiraterone treatment, cells were incubated during the 3 h with 10 μM of Abiraterone, with DMSO 1% being the control condition.

### 2.10. Adhesion Assay

Adhesion assays were carried out in 96-well plates; 5 × 10^4^ calcein (4 μM; Invitrogen, Carlsbad, CA, USA) stained cells were seeded per well in 100 μL of complete culture medium with 10% FBS. After 45 min of incubation, not attached cells were washed with PBS (×3). Attached cell fluorescence was measured using FLUOstar Optima (BMG labtech, Ortenberg, Germany) fluorimeter, in order to determine the percentage of attached cells.

### 2.11. Zebrafish Care and Breeding

Adult zebrafish (*Danio rerio*) were maintained in 30 L aquaria with a ratio of one fish per L of water, with 14:10 day/night cycle and a temperature of 28.5 °C, according to the standard procedures. Zebrafish embryos were obtained mating adult zebrafish in a proportion of two females to one male. All the procedures used in the experiments, fish care and treatment were performed in agreement with the Animal Care and Use Committee of the University of Santiago de Compostela and the standard protocols of Spain (Directive 2012-63-DaUE). Experimental protocols were approved by the Ethical Committee of the University of Santiago de Compostela (15010/2015/001). At the final point of the experiments, zebrafish embryos were euthanized by tricaine overdose.

### 2.12. Zebrafish Xenograft Assays and Image Analysis

Zebrafish embryos were collected from mating adults and incubated from 0 h to 48 h post fertilization (hpf) at 28.5 °C. At 48 hpf, embryos were anesthetized with 0.003% of tricaine (Sigma). Cell lines (MDA-MB-231, PLKO, SH2 and SH4) were incubated at 37 °C and 5% CO_2_ before the injection until they reached a confluence of 70%. Then they were trypsinized and concentrated at a rate of 1 million cells/Eppendorf/sample. After the concentration, all the conditions were dyed with the lipophilic marker DiI (according to the manufacturer protocol) and concentrated again separately in 10 µL of PBS (phosphate-buffered saline) with 2% of PVP (polyvinylpyrrolidone) to avoid cellular aggregation in each different condition.

Cell injection was carried out using borosilicate needles (1 mm O.D. × 0.75 mm I.D.; World Precision Instruments) and a microinjector (IM-31 Electric Microinjector, Narishige, London, UK) with an output pressure between 26 and 34 kPa and 30 ms of injection time per injection. Between 200–300 labeled cells were injected into the circulation of the embryo (duct of Cuvier). After the xenotransplantation, embryos were incubated during the 6 days post-injection (dpi) at 34 °C in 30 mL Petri dishes with salt dechlorinated tap water (SDTW). For testing Abiraterone activity, compound was diluted in DMSO and added to the water at a final concentration of 1 µM (selected in accord with the toxicity assays). Imaging of the injected embryos were performed at different time points during the incubation (1, 4 and 6 dpi) using a fluorescence stereomicroscope (AZ-100, Nikon, Melville, NY, USA) in order to measure the proliferation capacity of cells in each condition tested.

Quantifish software was used to quantify the proliferation of the injected cells by means of fluorescence of the labeled cells in each condition in the region of the caudal hematopoietic tissue (CHT) of the embryos, where the cells metastasize. Each of the images provided at different time points were measured using the software to obtain the number of positive pixels above a certain threshold and the intensity of the fluorescence. With these parameters, a value of integrated density is obtained allowing the researcher to compare different times between images and reaching a proliferation ratio.

### 2.13. Mouse Xenograft

For this study, SCID beige female mice (RRID: IMSR_CRL:250) were obtained from the Barcelona Biomedical Research Park (PRBB, Barcelona, Spain). Mice were housed and maintained under specific conditions of absence of pathogens and following the institutional guide approved by the committee for the use and care of animals, in SPF facilities of the Center for Research in Molecular Medicine and Chronic Diseases belonging to the University of Santiago de Compostela (ES150780275701). All the experimental procedures carried out were approved by the ethics committee of the University of Santiago de Compostela (15010/2015/001) and were designed and carried out by research personnel in possession of the corresponding accreditations marked by the guidelines of the Federation of European Associations for Laboratory Animal Science (FELASA) and included in current regulations.

For the establishment of the xenograft model, mice were anesthetized with a 2% isofluorane/oxygen mixture and either 2 × 10^6^ MDA-MB-231-PLKO or their respective TIMP1 silencing cell variant SH2 were injected into the mammary fat pad of the mice resuspended in a final volume of 60 µL of DMEM culture medium supplemented with 30% of Matrigel Matrix (Corning, NY, USA). Experimental groups: n_replica_ = 3 mice per group; n_total_ = 9 mice per group.

For tumor development monitoring, mice were followed-up weekly with RediJect 2-DG-750 Probe Standard Kit (Perkin Elmer, Walthan, MA, USA). Briefly, 100 μL of the reagent was injected intraperitoneally and image acquisition was performed after 3 h using the system IVIS (Xenogen Corporation) and Living Image software 4.2 (Xenogen Corporation, Alameda, CA). Mice were euthanized with carbon dioxide and verified by cervical dislocation. During mice necropsy, tissues from lymph nodes, lung and liver were collected, fixed in 40% formalin and included in paraffin. Subsequently, hematoxylin-eosin staining was performed to determine the presence of tumor cells using Mayer’s hematoxylin for 4 min and 0.2% eosin for 1 min. Immunohistochemistry was carried out to detect CK AE1/AE3 in the cases where it was necessary to confirm the presence of tumor cells. Hematoxylin-eosin and immunohistochemistry analysis were performed in the Pathology Department of the University Clinical Hospital of Santiago de Compostela.

### 2.14. Statistical Analysis

The statistical analyses were carried out using the software SPSS 22 for Macintosh (IBM Software Group, Chicago, IL, USA), Excel 2011 for Macintosh (Microsoft Corporation, Redmond, WA, USA) and GraphPad Prism 7.0 for Windows (GraphPad Softwares Inc., San Diego, CA, USA).

The expression comparisons of the markers analyzed between groups of patients and controls were carried out by means of two-sided Mann–Whitney U-test and *p*-values for each marker were adjusted by FDR (false discovery rate) test. This test was also used to compare the results of the in vitro assays. The outliers for each marker in the control and patient group were identified using Turkey’s method. Comparisons between groups in the in vivo tests with zebrafish were carried out using a two-tailed *t*-test analysis to determine the existence of significant differences. In a first step, the outliers of the data series were identified using the ‘identify outliers’ function of GraphPad software (ROUT Method).

Fisher test (two-sided) was used to determine the association between the clinico-pathological features and the levels of the CTC-markers grouped as high and low based on the cut-off which classifies the 30% of patients as high levels and the 70% as low levels (percentile 70). For analyzing the diagnostic accuracy of each of the markers, receiver operating characteristic (ROC) curves were used. Besides, a logistic model was generated combining the different markers to find the best combination to discriminate patients and healthy volunteers. Survival analyses were carried out by means of Kaplan–Meier and Cox regression analyses. For the survival analyses, the levels of CTC-markers were grouped as high/low as previously described. Overall (OS) and progression-free (PFS) survivals were calculated as the time between blood sample collection and patients’ progression/death or last disease control. For correlation analyses, continuous variables were evaluated using the Pearson correlation coefficient (two-sided).

For all the analyses, a probability lower than 5% was accepted as significant (*p* < 0.05).

## 3. Results

### 3.1. Patient Characteristics

A total of 32 patients treated for TNBC cancer were enrolled for this study between 2014 and 2017. Patient characteristics are shown in Table 1. The majority of tumors were stage IV (71.9%) and of new diagnosis (75%, including stage III and IV tumors). Besides, 17 out of the 23 metastatic patients (73.9%) had visceral metastasis, while 4 (17.4%) showed both visceral and bone dissemination at sample collection. On the other side, the 71.8% had previous surgery and the 15.6% were not naive for antitumor therapies. Regarding the patient’s evolution, it is important to mention that the median PFS and OS since blood sample collection were 12.4 and 18.4 months, respectively.

### 3.2. CTC Enumeration Correlates with a More Aggressive Disease

**Figure 1:**
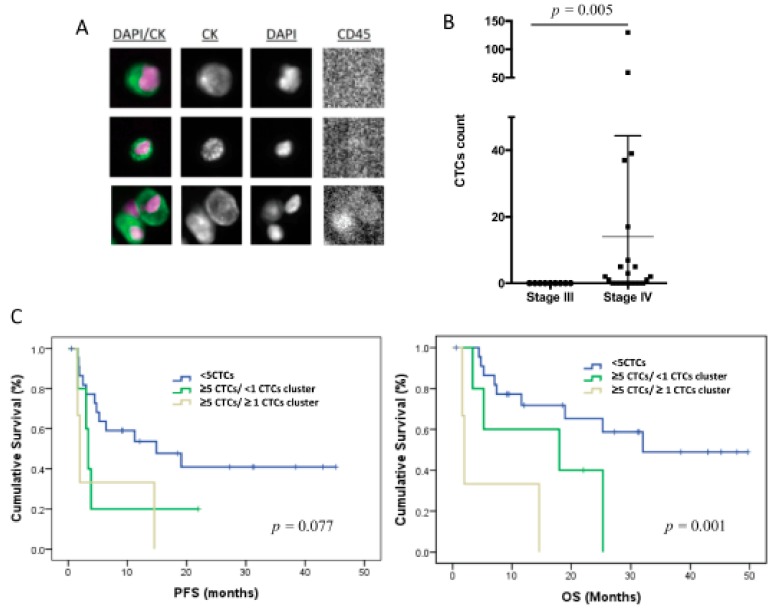
CTC enumeration by CellSearch system in TNBC patients (*n* = 31). (**A**) CTC images obtained using CellSearch system (round-oval, DAPI^+^, CD45^−^ and CK^+^ cells were considered as CTCs). (**B**) CTC count in stages III and IV TNBC patients using CellSearch system; *p*-value was calculated according to two-sided Mann–Whitney test. (**C**) Kaplan–Meier analysis for PFS and OS grouping patients according to the CTC count and the presence of CTC clusters; *p*-values were calculated using log-rank test.

The CTCs levels in 7.5 mL of peripheral blood were evaluated using CellSearch technology in 31 patients of the total cohort of 32 patients included in the study. Therefore, only epithelial CTCs expressing EpCAM and CKs were identified (Figure 1A). These cells were found in 14 patients (42%), in a range between 1 and 130 cells (Figure 1B), and 3 of the patients also showed CTC clusters.

Importantly, all the patients with positive CTC count were metastatic at sample collection, with eight (26%) of these patients showing ≥5 CTCs. No correlation was found between the CTC number and other clinicopathologic characteristics. However, patients with a number of CTCs ≥5 (*n* = 8) showed significantly poorer PFS and OS, in comparison with those with <5 CTCs (*n* = 23) (*p* = 0.033 and *p* = 0.006, respectively, according to log-rank test) (Table 2), evidencing the prognosis value of the EpCAM^+^ CTC population in our cohort of TNBC patients. Of note, the presence of CTC clusters in patients with CTCs ≥5 was also associated with poorer survival rates (*p* = 0.077 for PFS and *p* = 0.001 for OS, according to log-rank test) (Figure 1C, Table 2).

It is important to mention that when only patients with stage IV tumors at sample collection were included in the survival analyses, the same prognostic impact trend was observed in those patients with ≥5 CTCs or CTC clusters, although the statistical significance was lower because of the cohort size reduction (Table S2). In this sense, although there is scientific interest of these results about the prognostic value of the CTC count, they should be interpreted taking into account the low size of the unfavorable groups of patients.

### 3.3. CTCs from TNBC Patients Are Characterized by High Cell Plasticity

**Figure 2:**
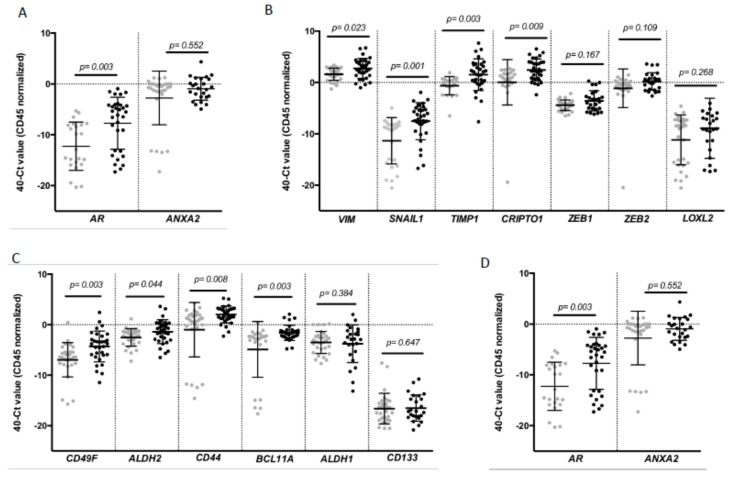
Gene expression profile of CTCs from patients with TNBC. Differential expression levels of genes involved in (**A**) epithelial (*CDH1*, *EPCAM*); (**B**) mesenchymal (*VIM*, *SNAIL1*, *TIMP1*, *CRIPTO1*, *ZEB1*, *ZEB2*, *LOXL2*); (**C**) stem cell features (*CD49F*, *ALDH2*, *CD44*, *BCL11A*, *ALDH1* and *CD133*) and (**D**) hormone regulation and this tumor aggressiveness *(AR*, *ANXA2*) in CTCs from TNBC patients compared to the background signal associated with the unspecific immunoisolation found in healthy controls. Gray symbols represent the gene expression levels in the group of healthy controls (*n* = 30), while black symbols are those corresponding to TNBC patients (*n* = 32). *p* values were calculated using two-sided Mann–Whitney U-test. FDR was employed to adjust these *p* values.

To complement the CTC enumeration using CellSearch system we characterized the CTC population in our cohort of patients by RT-qPCR after the immunoisolation of EpCAM^+^ CTCs as we previously described in other tumors [18,19]. For that analysis, peripheral blood samples from 30 healthy donors were used as reference control of the non-CTC-related expression of selected genes. We first confirmed the presence of an additional circulating population in patients in comparison to controls by means of *GAPDH* and *CD45* expression (Figure S1). Second, a further expression profile, including a panel of genes related to EMT, stemness phenotype and breast cancer aggressiveness, was carried out. After analyzing all the samples, we found a CTC population characterized by the expression of epithelial markers such as E-cadherin (*CDH1*) and *EPCAM* in consonance with the isolation strategy. However, these cells also differentially expressed genes associated with mesenchymal and more malignant features, such as *VIM*, *SNAIL1*, *TIMP1* and *CRIPTO1*, and stemness markers such as *CD49F*, *ALDH2*, *CD44* and *BCL11A* (Figure 2).

Of note, the levels of some these genes were clearly correlated. Thus, high levels of *VIM* were not only associated with high levels of EMT promoters such as *TIMP1* or *SNAIL1* and stem-like markers such as *CD44*, *ALDH2* and *CD49F,* but also with *EPCAM* and *CDH1*. Besides, all stem markers were strongly correlated (Table S3).

On the other side, after performing a ROC analysis, most of these genes presented an area under the ROC curve (AUROC) greater than 0.66 with significant *p*-values (Table 3) to discriminate patients from healthy controls, validating their utility to detect the presence of disseminated disease.

**Figure 3:**
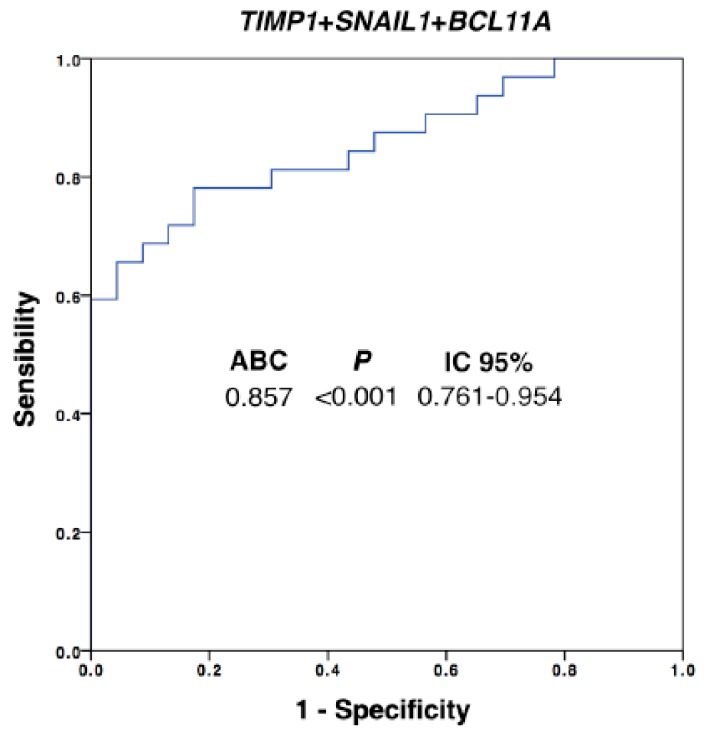
Logistic regression ROC model combining *TIMP1*, *SNAIL1* and *BCL11A* expression levels for patients’ discrimination. Equation: Ln(odds) = 2.02 + (0.31 × *TIMP1*_expression_) + (0.05 × *SNAIL*_expression_) + (0.55 × *BCL11A*_expression_); AUROC, area under the ROC curve; CI, confidence interval. Healthy controls, *n* = 30; patients, *n* = 32.

In addition, the diagnostic power of our approach was improved with respect to the use of individualized markers when different ones were combined. We performed a multivariate analysis by using binary logistic regression for the ROC curves with all the differential markers. Thus, after this analysis, the best panel to detect disseminated disease was obtained when *TIMP1*, *SNAIL1* and *BCL11A* were combined (Figure 3, AUROC 0.857, *p* = 0.001). Importantly, with this model we were able to determine the presence of disseminated disease in 60% of the patients with 100% of specificity (using a cut-off value of 0.756), in comparison with the 42% of patients classified as positive for CTCs using the CellSearch system.

On the other hand, when we compared the expression level of the analyzed CTC markers and the cell count obtained by CellSearch, we observed that patients with ≥5 CTC count showed significantly higher levels of *CDH1* (*p* = 0.018), *EPCAM* (*p* = 0.008) and *TIMP1* (*p* = 0.025), evidencing that epithelial CTCs identified by the CellSearch system express these three markers, as was expected, at least for the two epithelial markers (Figure S2), since the two isolation methods employed for both the enumeration and the gene expression profiling are based on EpCAM protein expression.

In addition, to determine the evolution of the markers during the dissemination process, we analyzed the panel identified in the population of CTCs in samples from the primary tumor, metastasis and healthy tissue obtained at diagnosis or surgery in 19 of the 32 patients. Although we only could perform this analysis in part of the patients, a significant increase in the expression levels of *EPCAM*, *SNAIL1*, *CD44*, *CDH1*, *TIMP1* and *BCL11A* was found in the primary tumor and metastases with respect to nontumoral tissue, reinforcing their possible role during both the tumor formation and spread (Figure S3A).

### 3.4. The CTC Expression Signature Predicted the Patients’ Outcome

The association between the CTC expression profile and the patient’s clinico-pathological characteristics were also addressed, grouping the gene expression levels as high/low based on the percentile 70. After this analysis, high levels of *CD49F* were associated with the presence of metastasis (*p* = 0.024), while nonmetastatic patients showed low levels of *AR* (*p* = 0.035) and *TIMP1* (*p* = 0.014), according to Fisher’s test (Table S4).

In order to determine the prognostic value of the markers present in the CTC population of our cohort of TNBC patients, a Kaplan–Meier survival analysis was carried out. For that, the expression levels of the markers were grouped as high or low as previously described. Importantly, increased expression of almost all markers was associated with worse OS, with this association being statistically significant for *CD49F*, *ALDH2*, *CD44*, *GAPDH* and *TIMP1* (Table S5). Besides *CD49F*, *GAPDH* and *TIMP1* showed statistically significant impact to predict the PFS (Table S5, Figure S3B). Of note, Cox regression analysis (Table 4) also evidenced that patients with high levels of *CD49F*, *TIMP1*, *GAPDH*, *ALDH2* or *CD44* presented 3 to 5 times higher risk of death than those with low levels. On the other hand, patients with high levels of *CD49F*, *TIMP1* or *GAPDH* showed 3 to 4 times higher risk of recurrence than patients with low levels of these markers (Table 4).

### 3.5. CTC Profiling Helps to Identify Therapeutic Targets such as AR and TIMP1

Once the high cell plasticity CTCs from TNBC patients and their prognostic impact was evidenced, we selected two markers that were significantly increased for further in vitro/vivo characterization, AR and TIMP1. We chose TIMP1 for a functional characterization because of its relevance for EMT and cancer progression in other breast cancer subtypes [20]. TIMP1 expression was knocked-down in MDA-MB-231 cell line, since this cell line nicely represents TNBC and expresses high levels of the marker. After stable shRNA transfection, we observed a significant reduction in TIMP1 levels both at mRNA and protein levels in the silenced cell lines (SH2 and SH4) in comparison with the control (PLKO) (Figure S4A) (*p* = 0.02 and *p* = 0.015, respectively). Of note, after addressing the proliferation, clonogenicity, adhesion and invasion capacity of the TIMP1 knock-down cells, a decrease of proliferation (*p* = 0.014 and *p* = 0.018 in SH2 and SH4, respectively) and colony formation (*p* = 0.045 and *p* = 0.06 in SH2 and SH4, respectively) together with an increase of adhesion to collagen (*p* = 0.028 and *p* = 0.028 in SH2 and SH4, respectively) were observed (Figure S4C–E).

**Figure 4:**
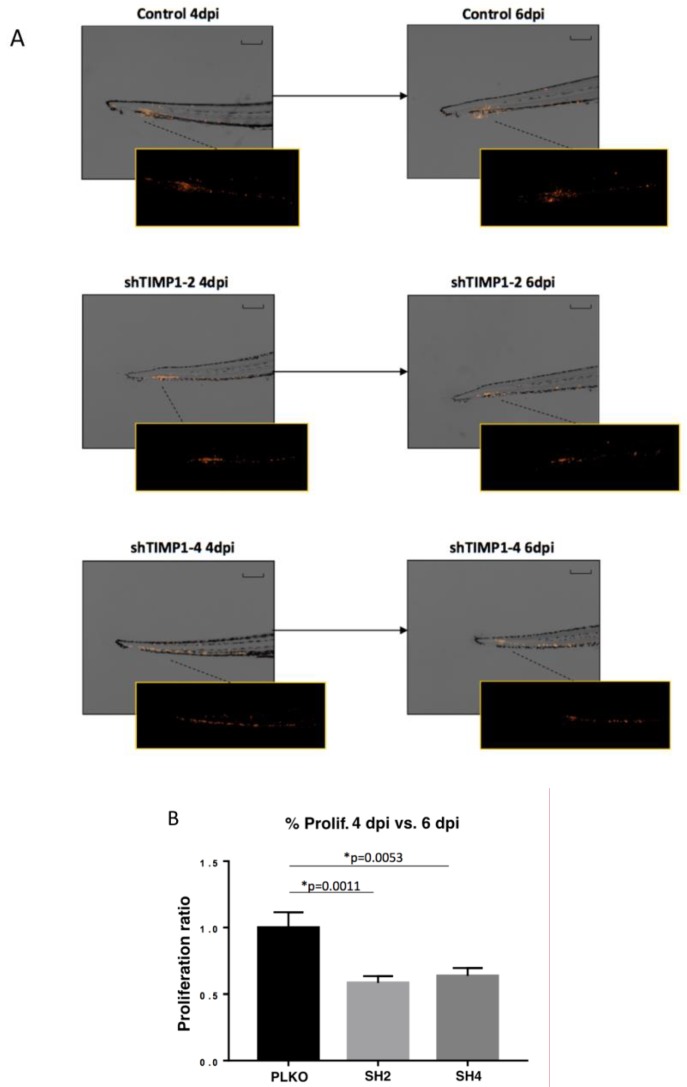
Effect of *TIMP1* knock-down in MDA-MB-231 proliferation in zebrafish embryos. (**A**) Representative images of the injected embryos with the different conditions of the cell line at 4 dpi and 6 dpi compared to 1 dpi. The main images are a superposition of a fluorescence image and a bright field image of the same embryo. Fluorescence images are a magnification of the areas marked in the main image. Scale = 250 µm. (**B**) Normalized tumor growth at 4 dpi (left panel) and 6 dpi (right panel). (n_replica_ = 15 embryos/condition, n_total_ = 45 embryos/condition; two-tailed T test; * *p* < 0.05; dpi: days post-injection).

We also used zebrafish embryos and mouse ortho-xenografts to analyze the in vivo effect of *TIMP1* down-regulation on our model cell line. Therefore, DiI-labeled MDA-MB-231 variants (SH2, SH4 and PLKO) were injected into the circulation of 48 hpf zebrafish embryos. After injection, the incubation of zebrafish embryos was carried out for 6 days at 34 °C. Our findings evidenced a significant reduction of proliferation in the TIMP1-knocked cells compared to the control cell line (Figure 4A,B) at 4 dpi and 6 dpi (*p* = 0.001 and *p* = 0.005 respectively, according to two-tailed *t*-test).

**Figure 5:**
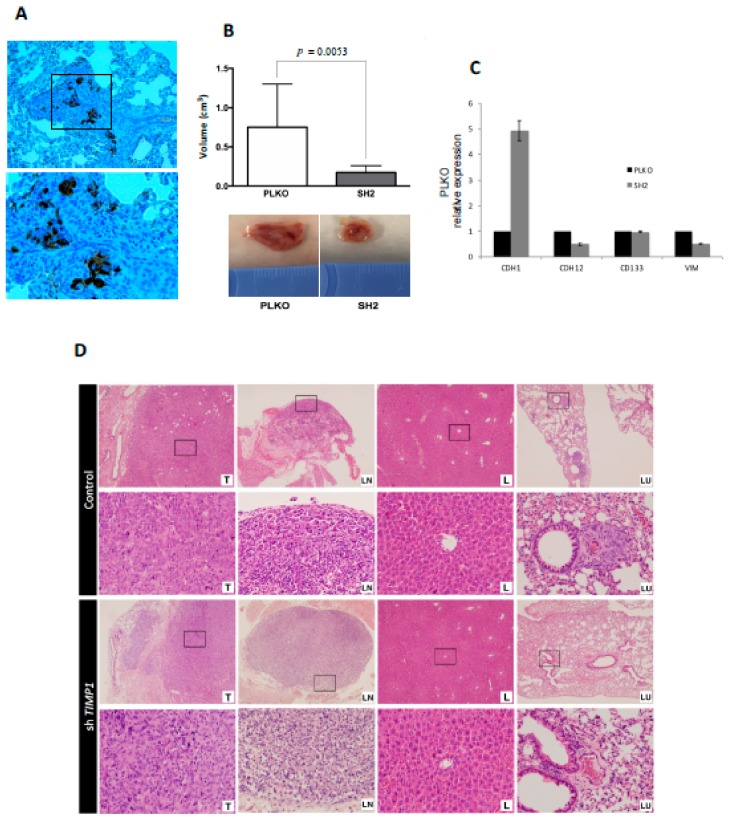
Impact of *TIMP1* knock-down in MDA-MB-231 proliferation and dissemination in a murine orthotopic model. (**A**) CKs (EA1/EA3) expression in the lung tissue of the control group, confirming the presence of lung metastasis. The image was taken at 20× and respective magnification (square) at 40×; (**B**) Mean tumor volume in PLKO and SH2 (n_replica_ = 3 mice/condition, n_total_ = 9 mice/condition; two-sided Mann–Whitney U-test; *p* = 0.0053) after sacrifice (upper image). Representative images of tumor volume in both experimental groups (down image); (**C**) *ECAD* (*CDH1*), *NCAD* (*CDH12*), *CD133* and *VIM* expression levels in primary tumors generated by PLKO and SH2 cell variants (*n* = 2); (**D**) Representative images of hematoxylin-eosin staining of the different mouse tissues after the orthotopic injection of the control cell line (PLKO) and the *TIMP1* silenced one (SH2). T: Primary tumor; LN: Axillary lymph node; L: Liver; LU: Lung. 4× images and their respective magnifications (square) at 20×.

The same impact on tumor proliferation was observed when the silenced and the control cells were injected into the mammary fat pad of SCID mice. For this assay, only the SH2 was analyzed because it showed the most significant effects in all functional analyses. Tumors grew after 3 weeks post-injection (Figure 5A) but SH2 showed significantly lower volume (70% less than control) at the end-point (Figure 5B).

Interestingly, nodal dissemination was detected only in 33% of mice injected with silenced cells, while all control mice showed lymph nodes affectation. Even more importantly, mice injected with the silenced cells never presented lung metastases, while all mice injected with the control cells developed lung dissemination (Figure 5D). These results indicated the impact of TIMP1 on the proliferative and disseminative behavior of our TNBC model.

In addition, to better characterize the molecular pathways mediating the role of TIMP1 on cell dissemination, we analyzed the gene expression levels of cell plasticity markers (E-cadherin, N-cadherin, CD133 and Vimentin) after TIMP1 down-regulation, finding a reduction of N-cadherin and CD133 and an increment of E-cadherin levels in both the tumor cell lines and the tumors generated in the mice model (Figure S4B and Figure 5C). These data suggest that TIMP1 is implicated in the acquisition of a more mesenchymal and stem-like phenotype.

Finally, because of the high expression of AR in CTCs from TNBC patients, we explored the effect of pharmacological inhibition of the AR pathway, which is a common therapeutic strategy in AR-dependent tumors such as prostate cancer [21]. To that end, we treated the MDA-MB 231 model cell line with Abiraterone, which inhibits androgen biosynthesis by CYP17 blockage. Interestingly, AR is highly expressed in some TNBC tumors but its role in tumorigenesis and metastasis of this BC subtype has not been well described [22]. Importantly, after treatment, MDA-MB-231 cells showed a significant decrease of cell proliferation in both attachment and suspension conditions in vitro and also when these cells were injected in zebrafish embryos (Figures S5 and S6).

All together, these results evidenced that CTC characterization is a valuable tool to identify not only prognostic markers but also molecules with interest as therapeutic targets to block the tumor growth and dissemination in TNBC.

## 4. Discussion

Hematogenous dissemination of tumor cells and subsequent metastatic formation in distant organs represent the leading cause of death in cancer patients [8]. In recent years, evidence of the prognostic relevance of CTCs in different tumor types such as breast cancer has been well described [23,24,25]. Besides, CTCs have special molecular characteristics, which are key to the formation of metastatic lesions, and allow them to successfully invade, survive in circulation and extravasate in remote locations [10].

TNBC is the most aggressive breast cancer subtype with a high metastatic rate, associated with an important molecular heterogeneity and absence of efficient targeted therapies [2]. Therefore, the advance in the knowledge of the CTC population in these patients can provide useful information to improve TNBC clinical management. In fact, our group recently characterized the biology of a TNBC patient through the generation of a CTCs-derived xenograft (CDX), providing valuable information regarding the molecular signatures altered in this patient [26]. Following this line of work, in the present study we quantified and characterized the EpCAM^+^ CTC population from a cohort of patients diagnosed with advanced or locally advanced TNBC. For that we used two strategies, the CellSearch system for enumeration and a combination of immunoisolation and gene expression analysis to molecularly characterize the CTC population.

Importantly, 42% of all analyzed patients were positive for CTCs (at least 1 CTCs) using CellSearch system, with all the positive cases being of metastatic patients. The percentage of CTC detection described in other studies varies between 16% and 73% depending on the analytic methods used and the tumor stage [5,7]. Thus, for example, Zhang et al. analyzed the levels of peri-operative CTCs using CellSearch technology in 286 cases of stages I, II and III TNBC, finding CTCs in the 23%, 37% and 56%, respectively, for each stage [7]. In a cohort of 102 patients with stage IV TNBC, Magbanua et al. described a detection rate (CTCs ≥5) with the CellSearch system before starting chemotherapy of 44%, and 15 days after starting treatment this percentage dropped to the 33%. These data together with our results evidenced the need for alternative techniques to improve the CTC detection, since a high percentage of metastatic patients are negative for CTCs using the CellSearch system.

Therefore, to better explore the potential of CTCs in the context of TNBC, we employed a strategy successfully applied by our group on different tumor types to get insights on the molecular profile of CTCs [18,19]. The immunoisolation of the EpCAM^+^ CTCs and their posterior gene expression profiling allowed us to detect a higher number of patients positive for CTCs (60%) in comparison with the CellSearch system and also to identify the existence of an important cell plasticity phenotype in the circulating cell population, that is likely to represent a key factor for the efficient spread characterizing TNBC tumors. The expression of E-cadherin and EpCAM on the CTCs isolated from our cohort of patients, previously described in other cohorts of TNBC patients [26,27], demonstrates the epithelial origin of these cells. Of note, the activity of epithelial proteins is especially important during the last stages of colonization where tumor cells have to proliferate to re-establish a new tumor focus [28,29]. On the other hand, the expression of genes such as *VIM*, *SNAIL1*, *TIMP1*, *CRIPTO1*, *CD49F*, *ALDH2*, *CD44* and *BCL11A* in CTCs from our TNBC population suggests the importance of the presence of mesenchymal and stem cell markers that might contribute to the survival of these cells in the blood. In fact, numerous studies have shown that transition from epithelial to mesenchymal characteristics induces the appearance of stem cell properties in tumor cells of epithelial origin, being these hybrid or intermediate phenotypes linked to greater metastatic capacity [27,28]. Besides, works in which the circulating tumor population has been characterized revealed the appearance of these intermediate phenotypes, with a clear impact on the survival and the colonization efficiency of these cells [18,19,29] in agreement with the present study. In fact, it is well known that during the process of intravasation and migration into the bloodstream cell plasticity is important, allowing CTCs to survive under an extremely adverse environment, overcome the lack of O_2_, avoid the activation of anoikis and also the immune cells’ recognition [16,30,31,32,33]. In line with this idea, we found higher levels of *TIMP1*, *AR* and *CD49F* in CTCs from metastatic patients than in non-metastatic, which could be associated with an increment in the CTC number but also with an increment of these genes expression along with the disease evolution.

In addition, our results evidenced that presence of EpCAM^+^ CTCs with greater phenotypic plasticity is associated with a worse evolution of the disease, providing additional prognostic information to the oncologist for the clinical management of these patients. In this sense, it is important to highlight that no clinical variables analyzed in the present study showed prognostic value in our patient cohort, probably because it was a clinically heterogeneous cohort of an already poor outcome, due to the fact that 72% were metastatic TNBC at the sample collection. Our results also reinforce the relevance of applying versatile enrichment techniques to the isolation of nonstrict epithelial phenotypes, although the EpCAM based isolation allowed us to reach very acceptable detection rates, especially when plasticity markers were included.

On the other hand, one of the objectives of this work was the identification of potential therapeutic targets through the characterization of the CTCs of TNBC patients. Therefore, from the signature of markers present in the circulating population we selected two, the AR and TIMP1, for a further characterization through in vitro/vivo functional studies.

The androgen receptor acts as a transcription factor for numerous genes after androgen binding (testosterone and dihydrotestosterone). This receptor is overexpressed in 12%–36% of TNBC [34,35], called LAR (luminal subtype with expression of AR). This expression seems to be a predictive factor of the poor response to chemotherapy based on taxanes and anthracyclines, since patients with high expression has the lowest rates of complete response compared to the rest of TNBC [36]. Also, a very recent study demonstrated the association between the presence of AR^+^ CTCs and the formation of bone metastases in patients with ER^+^ breast tumors [37]. Our study evidenced for the first time the presence of high levels of AR in CTCs from TNBC suggesting its potential a therapeutic target to kill these cells. After treating MDA-MB-231 with Abiraterone, which impairs the androgen pathway at different levels, a significant decrease of cell proliferation was observed in vitro and in zebrafish embryos. Although we did not further explore the molecular mechanisms behind this effect on our cell line model, our results suggest the therapeutic interest of this pathway for the treatment of TNBC, with CTCs representing a very useful tool to determine the status of this receptor in metastatic patients as occurs in metastatic prostate cancer patients [38,39].

We also focused our attention on TIMP1. This protein was identified two decades ago and initially characterized as an endogenous MMP (matrix metalloproteinase) inhibitor [40], being broadly recognized for its role remodeling the extracellular matrix [41]. TIMP1 overexpression has been described in different types of human cancer, including prostate cancer [42], lung cancer [43], melanoma [44], glioblastoma [45] and breast cancer [46]. In TNBC tumors, TIMP1 is also overexpressed, as we found in our cohort of patients, with this high expression being associated with a worse prognosis of the disease [20]. However, although TIMP1 has been described as an interesting therapeutic target in several types of tumors, due to its role as an inhibitor of MMP9 and also for its role as a promoter of proliferation and angiogenesis, its implication in TNBC aggressiveness is not well defined. Here, we knocked down TIMP1 expression of MDA-MB-231 cell line and characterized the behavior of these cells in vitro and vivo, finding an effect on cell proliferation and adhesion. TIMP1-deficient cells showed a lower rate of proliferation in vitro as well as in vivo both in zebrafish embryos and in orthotopic xenografts in immunosuppressed mice. Of note, in the mouse models, TIMP1 downregulation strongly decreased cell proliferation and impaired the appearance of lung metastasis, supporting the potential role of this protein to favor TNBC growth and spread. In line with these observations, the characterization of MDA-MB-231 cell line after TIMP1 downregulation indicated a reduction of expression of mesenchymal markers. This result is compatible with a lower proliferative and disseminative capacity in vitro and in vivo, however, a further characterization of EMT would finally demonstrate this hypothesis. Additionally, PI3K, MEK, p38 and cyclin D1 activation has been described as result of TIMP1 activity in different breast cancer cells, including MDA-MB-231 [20,47], providing the molecular basis mediating the proliferation inhibition observed in our experimental models.

It is important to highlight that our study, although it counts with a limited patient population, reinforces the value of the CTC analysis in TNBC not only as a noninvasive prognostic tool but also for better understanding the molecular characteristics of this population during their travel through the bloodstream. In fact, as we already mentioned, TNBC is highly heterogeneous at a molecular level. Therefore, the real clinical value of the CTC-markers identified should be validated in a new, prospective, larger cohort, including patients with balanced tumor stage and with a broader characterization of their molecular subtype, to be able to better discriminate the association of the biomarkers with the pathologic profile of each tumor. Another limitation of the study is the fact that with our analytic approach, the under/overexpression of the different markers in the CTC population cannot be precisely estimated, and we can only associate high levels of the markers with a high CTC count. Despite these challenges, we successfully characterized the CTC population and validated this approach for the identification of molecular targets in vitro and in vivo. In fact, the study is novel in using a zebrafish model to characterize the role of TIMP1 and AR in TNBC behavior and to also translate the study of both markers to the CTC population in a cohort of TNBC.

## 5. Conclusions

Overall, with the present study we show that CTC count and, even more important, their molecular characterization constitutes a good alternative to obtain relevant information to establish new diagnostic, prognosis and monitoring options for patients with TNBC. This liquid-biopsy-based approach constitutes an interesting tool to explore the mechanisms leading to tumor spread of TNBC. In fact, we found cellular plasticity in CTCs from TNBC patients, in terms of expression of hybrid EMT and stem cell markers that can be associated with the poor prognosis and high aggressiveness of these tumors. Likewise, we reinforced the interest of two of the genes expressed in the CTC population, TIMP1 and AR, as molecules participating in TNBC proliferation promotion. All these data highlight the interest of addressing the characterization of CTCs as the most complete liquid biopsy to better understand the mechanisms promoting TNBC aggressiveness, since these tumors are extremely complex to be characterized only by means of primary tumor analyses.

## Figures and Tables

**Table 1:** Clinico-pathologic characteristics of the cohort of patients with triple-negative breast cancer (TNBC).

Age (Years)	Mean (Range)		
	58.5 (33–80)		
Stage *	***n* (%)**	**Previous Surgery**	***n* (%)**
**III**	9 (28.1%)	**Yes**	23 (71.8%)
**IV**	23 (71.9%)	**No**	9 (28.2%)
Status *		Treatment *	
**First Diagnosis**	24 (75.0%)	**Yes**	5 (15.6%)
**Recurrence**	8 (25.0%)	**No**	27 (84.4%)
**Metastasis location**		**Ki67 levels**	
**Visceral/bone**	4 (17.4%)	**Low**	4 (12.5%)
**Visceral**	17 (73.9%)	**High**	27 (84.4%)
**Unknown**	2 (8.7%)	**Unknown**	1 (3.1%)
**Histology**		**Disease evolution**	
**Ductal**	29 (90.6%)	**Progressions**	20 (62.5%)
**Lobulillar**	1 (3.1%)	**PFS (months)**	median (range)
**Metaplasic**	2 (6.3%)		12.4 (0.5–45.2)
**Histology Grade**		**Survival**	
**3**	22 (68.7%)	**Deaths**	17 (53.1%)
**2**	10 (31.3%)	**OS (months)**	median (range)
			18.4 (0.5–45.2)

* Status at sample collection; PFS, progression free survival; OS, overall survival.

**Table 2:** Prognosis value of circulating tumor cell (CTC) levels in TNBC patients.

Marker	*n*	PFS (Months)		OS (Months)	
		Mean (95% CI)	*p*	Mean (95% CI)	*p*
**CTCs levels**					
<5 CTCs	23	22.7 (14.3–31.1)	**0.033**	32.3 (23.7–40.8)	**0.006**
≥5 CTCs	8	6.5 (1.6–11.4)		11.9 (4.8–19.1)	
**CTCs clusters**					
No (<1CTC cluster)	28	20.7 (13.2–28.3)	0.071	29.4 (21.7–37.1)	**0.001**
Yes (≥1CTC cluster)	3	6.04 (0.0–14.3)		6.04 (0.0–14.3)	
**CTCs levels/clusters**					
<5 CTCs	23	22.7 (14.3–31.2)		32.3 (23.7–40.8)	
≥5 CTCs and 0 CTCs cluster	5	6.8 (0.8–13.4)	0.077	15.5 (5.8–25.0)	**0.001**
≥5 CTCs and ≥1CTCs cluster	3	6.04 (0.0–14.3)		6.04 (0–14.3)	

PFS, progression free survival; OS, overall survival; CI, confidence interval; *p* values were calculated using log-rank test. The bold indicated the significance.

**Table 3:** Diagnostic value of CTC markers.

Marker	AUROC	*p*	95% CI
***EPCAM***	0.697	0.013	0.560–0.834
***AR***	0.758	0.001	0.633–0.884
***TIMP1***	0.764	0.001	0.636–0.891
***CRIPTO1***	0.727	0.004	0.595–0.858
***CDH1***	0.712	0.008	0.571–0.853
***VIM***	0.659	0.046	0.515–0.803
***CD49F***	0.755	0.001	0.624–0.887
***ALDH2***	0.648	0.063	0.502–0.795
***CD44***	0.696	0.014	0.554–0.837
***SNAIL1***	0.793	<0.001	0.673–0.912
***BCL11A***	0.766	0.001	0.637–0.894
***GAPDH***	0.665	0.027	0.520–0.811

AUROC, area under the ROC curve; CI, confidence interval.

**Table 4:** Univariate Cox regression analysis for CTC markers.

	Marker	HR (95% CI)	*p*
**OS**	*CD49F* (high vs. low)	5.12 (1.61–15.25)	0.006
	*ALDH2* (high vs. low)	3.12 (1.16–8.41)	0.024
	*CD44* (high vs. low)	3.70 (1.37–9.95)	0.010
	*TIMP1* (high vs. low)	5.12 (1.87–14.02)	0.001
	*GAPDH* (high vs. low)	5.18 (1.49–15.29)	0.004
**PFS**	*CD49F* (high vs. low)	3.33 (1.23–9.01)	0.018
	*TIMP1* (high vs. low)	3.86 (1.39–10.70)	0.004
	*GAPDH* (high vs. low)	3.56 (1.34–10.17)	0.014

OS, overall survival; PFS, progression free survival; HR, hazard ratio; CI, confidence interval; cut-off value to determine high and low expression was calculated based on percentile 70.

## Supplementary Materials

The following are available online at https://www.mdpi.com/2077-0383/9/2/353/s1, Figure S1: *GAPDH* and *CD45* expression in the CTCs enriched fraction from TNBC patients and healthy controls. (**A**) GAPDH expression levels, normalized by CD45, in the CTCs fraction of patients and controls, analysed by RT-q-PCR. (**B**) CD45 expression levels, in the CTCs fraction of patients and controls, analysed by RT-q-PCR. *p* values were calculated using two-sided Mann-Whitney U Test. C and D, ROC curve analysis to determine the diagnosis value of GAPDH and CD45, respectively. For all analyses, patients *n* = 32 and controls *n* = 30. Figure S2: *TIMP1*, *EPCAM* and *CDH1* expression levels in the CTCs fraction of patients and controls according to the CTCs count (CTCs ≥ 5 (*n* = 8) vs. < 5 (*n* = 23)). *p* values were calculated using two-sided Mann-Whitney U Test. Figure S3: (**A**) Gene expression profile of CTCs markers in normal, tumoral and metastatic tissue in TNBC patients (*n* = 19). Normal tissue (white boxes), tumoral tissue (light grey boxes), metastatic tissue (dark grey boxes). Paired Mann-Whitney U Test was used to calculate the *p* values. (**B**) Kaplan-Meier analysis for PFS and OS grouping patients according to the TIMP1 expression levels (high vs. low according to percentile 70). *p* values were calculated using Log-Rank test. For survival analyses, patients with high levels were 9 while patients with low levels were 23. Figure S4: In vitro effect of *TIMP1* knock-down on MDA-MB-231 cell line. (**A**) *TIMP1* expression levels analysed by RT-q-PCR (left) and Western blot (right) in the control (PLKO) and the *TIMP1* knocked-down cell lines (SH2 and SH4). (**B**) Expression levels of *ECAD* (*CDH1*), *NCAD* (*CDH12*), *CD133* and *VIM* in PLKO, SH2 and SH4 cells analysed by RT-q-PCR. C, D, E, Results of the cell proliferation, colony formation and adhesion to collagen assays, respectively. In all experiments *n* = 3. Two-sided Mann-Whitney U Test was used to calculate the *p*-values. Figure S5: In vitro impact of Abiraterone treatment on MDA-MB-231 behavior. (**A**) Proliferation assay. (**B**) Colony formation in agar assay. (**C**) Migration assay. Two-sided Mann-Whitney U Test was used to calculate the *p*-values. Figure S6: In vivo proliferation of MDA-MB-231 after injection in zebrafish embryos in presence of Abiraterone. Normalized tumor growth at 4 dpi and 6 dpi (*n*_replica_ = 15 embryos/condition, *n*_total_ = 45 embryos/condition; two-tailed T test; * *p* < 0.05; dpi: days post-injection. Table S1: List of RT-qPCR assays employed in the study. Table S2: Prognosis value of CTCs levels in stage IV TNBC patients. Table S3. Correlation between the CTCs markers. Table S4: Association between the CTCs-markers and the metastasic status. Table S5. Prognosis value of markers identified on CTCs from TNBC patients.
