# Supplementary material for: Looking for a Better Characterization of Triple-Negative Breast Cancer by Means of Circulating Tumor Cells
Source: J Clin Med. 2020 Jan 27;9(2):353. doi: 10.3390/jcm9020353 (PMC7074553; doi:10.3390/jcm9020353)
Supplement: Supplementary file 1 [file jcm-09-00353-s001.zip › supp/Table S1.docx]

Table S1. List of RT-qPCR assays employed in the study.

| **Gene** | **Taqman assay** |
| --- | --- |
| *CD45* | Hs_00894734_m1 |
| *CDH1* | Hs_00170423_m1 |
| *EPCAM* | Hs_00158980_m1 |
| *VIM* | Hs_00958116_m1 |
| *ZEB1* | Hs_01566407_m1 |
| *ZEB2* | Hs_00207691_m1 |
| *LOXL2* | Hs_00158757_m1 |
| *SNAI1* | Hs_00195591_m1 |
| *ANXA2* | Hs_01561520_m1 |
| *TIMP1* | Hs_00171558_m1 |
| *CRIPTO1* | Hs_02339499_g1 |
| *AR* | Hs_00171172_m1 |
| *ALDH1* | Hs_00946916_m1 |
| *ALDH2* | Hs_01007998_m1 |
| *CD133* | Hs_01009250_m1 |
| *CD49F* | Hs_01041011_m1 |
| *CD44* | Hs_01075861_m1 |
| *BCL11A* | Hs_01093197_m1 |
| *GAPDH* | Hs_99999905_m1 |
