# Supplementary material for: Looking for a Better Characterization of Triple-Negative Breast Cancer by Means of Circulating Tumor Cells
Source: J Clin Med. 2020 Jan 27;9(2):353. doi: 10.3390/jcm9020353 (PMC7074553; doi:10.3390/jcm9020353)
Supplement: Supplementary file 1 [file jcm-09-00353-s001.zip › supp/Table S2..docx]

| **Marker** | **n** | **PFS (months)** | | **OS (months)** | |
| --- | --- | --- | --- | --- | --- |
|  |  | **mean (95% CI)** | **p** | **mean (95% CI)** | **p** |
| ***CTCs levels*** |  |  | |  | |
| Low (<5 CTCs) | 14 | 14.3 (4.9-23.6) | 0.313 | 27.6 (4.8-19.1) | 0.074 |
| High (≥5 CTCs) | 8 | 6.5 (1.6-11.4) |  | 11.9 (4.8-19.1) |  |
| ***CTCs clusters*** |  |  | |  | |
| No (0 CTCs cluster) | 19 | 13.6 (5.8-21.4) | 0.295 | 24.6 (14.4-33.8) | **0.015** |
| Yes (≥1CTCs cluster) | 3 | 6.04 (0-14.4) |  | 6.04 (0-14.4) |  |
| ***CTCs levels/ clusters*** |  |  |  |  |  |
| <5 CTCs | 14 | 14.2 (4.9-23.6) |  | 27.6 (16.1-38.9) |  |
| ≥5 CTCs and 0 CTCs cluster | 5 | 6.8 (0.1-13.5) | 0.490 | 15.4 (5.8-25.1) | **0.034** |
| ≥5 CTCs and ≥1CTCs cluster | 3 | 6.04 (0.0-14.4) |  | 6.04 (0.0-14.4) |  |

Table S2. Prognosis value of CTCs levels in patients with stage IV TNBC.

PFS; progression free survival; OS, overall survival; CI, confidence interval; P-values were calculated using Log-Rank test.
