# Supplementary material for: Looking for a Better Characterization of Triple-Negative Breast Cancer by Means of Circulating Tumor Cells
Source: J Clin Med. 2020 Jan 27;9(2):353. doi: 10.3390/jcm9020353 (PMC7074553; doi:10.3390/jcm9020353)
Supplement: Supplementary file 1 [file jcm-09-00353-s001.zip › supp/Table S3.docx]

Table S3. Correlation between the CTCs markers.

|  | | | | | | | | | | | | | |
| --- | --- | --- | --- | --- | --- | --- | --- | --- | --- | --- | --- | --- | --- |
|  | | ***CDH1*** | ***VIM*** | ***CD49F*** | ***EPCAM*** | ***ALDH2*** | ***CD44*** | ***SNAIL1*** | ***BCL11A*** | ***AR*** | ***TIMP1*** | ***CRIPTO1*** | ***GAPDH*** |
| ***CDH1*** | Pearson correlation | 1 | .443^*^ | .142 | .309 | .352^*^ | .335 | .295 | -.025 | -.041 | .287 | .184 | .332 |
|  | p (2-tailed) |  | .011 | .437 | .085 | .048 | .061 | .101 | .891 | .824 | .112 | .312 | .068 |
|  | N | 32 | 32 | 32 | 32 | 32 | 32 | 32 | 32 | 32 | 32 | 32 | 32 |
| ***VIM*** | Pearson correlation | .443^*^ | 1 | .649^**^ | .366^*^ | .951^**^ | .925^**^ | .668^**^ | -.047 | .231 | .722^**^ | .464^**^ | .550^**^ |
|  | p (2-tailed) | .011 |  | .000 | .040 | .000 | .000 | .000 | .800 | .204 | .000 | .008 | .001 |
|  | N | 32 | 32 | 32 | 32 | 32 | 32 | 32 | 32 | 32 | 32 | 32 | 32 |
| ***CD49F*** | Pearson correlation | .142 | .649^**^ | 1 | .237 | .615^**^ | .618^**^ | .583^**^ | -.008 | .567^**^ | .775^**^ | .472^**^ | .433^*^ |
|  | p (2-tailed) | .437 | .000 |  | .191 | .000 | .000 | .000 | .967 | .001 | .000 | .006 | .015 |
|  | N | 32 | 32 | 32 | 32 | 32 | 32 | 32 | 32 | 32 | 32 | 32 | 32 |
| ***EPCAM*** | Pearson correlation | .309 | .366^*^ | .237 | 1 | .367^*^ | .303 | .385^*^ | .156 | -.032 | .288 | .364^*^ | .351 |
|  | p (2-tailed) | .085 | .040 | .191 |  | .039 | .092 | .030 | .393 | .863 | .110 | .040 | .053 |
|  | N | 32 | 32 | 32 | 32 | 32 | 32 | 32 | 32 | 32 | 32 | 32 | 32 |
| ***ALDH2*** | Pearson correlation | .352^*^ | .951^**^ | .615^**^ | .367^*^ | 1 | .961^**^ | .621^**^ | -.050 | .230 | .719^**^ | .375^*^ | .568^**^ |
|  | p (2-tailed) | .048 | .000 | .000 | .039 |  | .000 | .000 | .786 | .205 | .000 | .034 | .001 |
|  | N | 32 | 32 | 32 | 32 | 32 | 32 | 32 | 32 | 32 | 32 | 32 | 32 |
| ***CD44*** | Pearson correlation | .335 | .925^**^ | .618^**^ | .303 | .961^**^ | 1 | .624^**^ | -.125 | .266 | .732^**^ | .349 | .650^**^ |
|  | p (2-tailed) | .061 | .000 | .000 | .092 | .000 |  | .000 | .497 | .141 | .000 | .050 | .000 |
|  | N | 32 | 32 | 32 | 32 | 32 | 32 | 32 | 32 | 32 | 32 | 32 | 32 |
| ***SNAIL1*** | Pearson correlation | .295 | .668^**^ | .583^**^ | .385^*^ | .621^**^ | .624^**^ | 1 | -.102 | .525^**^ | .677^**^ | .324 | .553^**^ |
|  | p (2-tailed) | .101 | .000 | .000 | .030 | .000 | .000 |  | .580 | .002 | .000 | .070 | .001 |
|  | N | 32 | 32 | 32 | 32 | 32 | 32 | 32 | 32 | 32 | 32 | 32 | 32 |
| ***BCL11A*** | Pearson correlation | -.025 | -.047 | -.008 | .156 | -.050 | -.125 | -.102 | 1 | .061 | -.012 | .091 | -.316 |
|  | p (2-tailed) | .891 | .800 | .967 | .393 | .786 | .497 | .580 |  | .740 | .947 | .620 | .083 |
|  | N | 32 | 32 | 32 | 32 | 32 | 32 | 32 | 32 | 32 | 32 | 32 | 32 |
| ***AR*** | Pearson correlation | -.041 | .231 | .567^**^ | -.032 | .230 | .266 | .525^**^ | .061 | 1 | .533^**^ | .039 | .157 |
|  | p (2-tailed) | .824 | .204 | .001 | .863 | .205 | .141 | .002 | .740 |  | .002 | .834 | .398 |
|  | N | 32 | 32 | 32 | 32 | 32 | 32 | 32 | 32 | 32 | 32 | 32 | 32 |
| ***TIMP1*** | Pearson correlation | .287 | .722^**^ | .775^**^ | .288 | .719^**^ | .732^**^ | .677^**^ | -.012 | .533^**^ | 1 | .426^*^ | .509^**^ |
|  | p (2-tailed) | .112 | .000 | .000 | .110 | .000 | .000 | .000 | .947 | .002 |  | .015 | .003 |
|  | N | 32 | 32 | 32 | 32 | 32 | 32 | 32 | 32 | 32 | 32 | 32 | 32 |
| ***CRIPTO1*** | Pearson correlation | .184 | .464^**^ | .472^**^ | .364^*^ | .375^*^ | .349 | .324 | .091 | .039 | .426^*^ | 1 | .411^*^ |
|  | p (2-tailed) | .312 | .008 | .006 | .040 | .034 | .050 | .070 | .620 | .834 | .015 |  | .022 |
|  | N | 32 | 32 | 32 | 32 | 32 | 32 | 32 | 32 | 32 | 32 | 32 | 32 |
| ***GAPDH*** | Pearson correlation | .332 | .550^**^ | .433^*^ | .351 | .568^**^ | .650^**^ | .553^**^ | -.316 | .157 | .509^**^ | .411^*^ | 1 |
|  | p (2-tailed) | .068 | .001 | .015 | .053 | .001 | .000 | .001 | .083 | .398 | .003 | .022 |  |
|  | N | 32 | 32 | 32 | 32 | 32 | 32 | 32 | 32 | 32 | 32 | 32 | 32 |
| * p<0.05 (two-tailed), ** p<0.01 (two-tailed). | | | | | | | | | | | | | |
