# Supplementary material for: Looking for a Better Characterization of Triple-Negative Breast Cancer by Means of Circulating Tumor Cells
Source: J Clin Med. 2020 Jan 27;9(2):353. doi: 10.3390/jcm9020353 (PMC7074553; doi:10.3390/jcm9020353)
Supplement: Supplementary file 1 [file jcm-09-00353-s001.zip › supp/Table S4.docx]

Table S4. Association between the CTCs-markers and the metastasic status.

| **Marker** | **N total** | **Non Metastatic**  **n** | **Metastatic**  **n** |  | |
| --- | --- | --- | --- | --- | --- |
|  |  |  |  | **OR (95% CI)** | **p** |
| ***CDH1*** |  |  |  |  | |
| Low | 23 | 8 | 15 | 0.23 (0.02 -2.22) | 0.383 |
| High | 9 | 1 | 8 |  |  |
| ***VIM*** |  |  |  |  | |
| Low | 23 | 7 | 16 | 0.65 (0.10- 3.97) | 1.000 |
| High | 9 | 2 | 7 |  |  |
| ***CD49F*** |  |  |  |  | |
| Low | 23 | 9 | 14 | 0.84 (0.60-1.27) | **0.024** |
| High | 9 | 1 | 8 |  |  |
| ***EPCAM*** |  |  |  |  | |
| Low | 23 | 8 | 15 | 0.23 (0.02 -2.22) | 0.383 |
| High | 9 | 1 | 8 |  |  |
| ***ALDH2*** |  |  |  |  | |
| Low | 23 | 7 | 16 | 0.65 (0.10-3.97) | 1.000 |
| High | 9 | 2 | 7 |  |  |
| ***CD44*** |  |  |  |  | |
| Low | 23 | 7 | 16 | 0.65 (0.10-3.97) | 1.000 |
| High | 9 | 2 | 7 |  |  |
| ***SNAIL1*** |  |  |  |  | |
| Low | 23 | 7 | 16 | 0.65 (0.10-3.97) | 1.000 |
| High | 9 | 2 | 7 |  |  |
| ***BCL11A*** |  |  |  |  | |
| Low | 23 | 4 | 19 | 5.93 (1.08-32.51) | 0.075 |
| High | 9 | 5 | 4 |  |  |
| ***AR*** |  |  |  |  | |
| Low | 23 | 10 | 13 | 0.73 (0.50-1.07) | **0.035** |
| High | 9 | 1 | 8 |  |  |
| ***TIMP1*** |  |  |  |  | |
| Low | 23 | 9 | 14 | 0.61 (0.44-0.84) | **0.014** |
| High | 9 | 0 | 9 |  |  |
| ***CRIPTO1*** |  |  |  |  | |
| Low | 23 | 6 | 17 | 1.14 (0.26-7.52) | 0.685 |
| High | 9 | 3 | 6 |  |  |
| ***GAPDH*** |  |  |  |  | |
| Low | 23 | 8 | 15 | 0.61 (0.10-3.73) | 0.689 |
| High | 9 | 2 | 7 |  |  |

Cut-off value to determine high and low expression was calculated based on percentile 70; Fisher’s test (two-sided) was used to determine the p-values; OR; odd ratio; CI, confidence interval.
