# Supplementary material for: Looking for a Better Characterization of Triple-Negative Breast Cancer by Means of Circulating Tumor Cells
Source: J Clin Med. 2020 Jan 27;9(2):353. doi: 10.3390/jcm9020353 (PMC7074553; doi:10.3390/jcm9020353)
Supplement: Supplementary file 1 [file jcm-09-00353-s001.zip › supp/Table S5.docx]

| **Marker** | **n** | **Overall survival (OS)** | | **Progression free survival (PFS)** | |
| --- | --- | --- | --- | --- | --- |
|  |  | **mean (95% CI)** | **p** | **mean (95% CI)** | **p** |
| ***CDH1*** |  |  | |  | |
| Low | 23 | 28.89 (20.44 - 37.35) | 0.210 | 21.69 (13.34 -30.05) | 0.133 |
| High | 9 | 18.94 (6.33 - 31.55) |  | 9.23 (2.40 - 16.06) |  |
| ***VIM*** |  |  | |  | |
| Low | 23 | 30.22 (21.46 - 38.99) | 0.087 | 21.14 (12.84 - 29.44) | 0.209 |
| High | 9 | 15.05 (8.29 - 21.80) |  | 9.17 (1.94 - 16.40) |  |
| ***CD49F*** |  |  | |  | |
| Low | 23 | 31.57 (23.39 - 39.75) | **0.002** | 23.30 (15.00 - 31.61) | **0.011** |
| High | 9 | 9.42 (5.04 - 13.81) |  | 4.12 (2.64 - 5.61) |  |
| ***EPCAM*** |  |  | |  | |
| Low | 23 | 30.40 (21.88 - 38.93) | 0.057 | 20.26 (12.37 - 28.14) | 0.220 |
| High | 9 | 15.19 (5.13 - 25.25) |  | 12.11 (1.24 - 22.97) |  |
| ***ALDH2*** |  |  | |  | |
| Low | 23 | 31.70 (22.78 - 40.63) | **0.017** | 22.77 (14.23 - 31.31) | 0.072 |
| High | 9 | 14.23 (7.89 - 20.57) |  | 7.16 (2.72 - 11.60) |  |
| ***CD44*** |  |  | |  | |
| Low | 23 | 32.12 (23.45 - 40.79) | **0.006** | 22.75 (14.21 - 31.30) | 0.077 |
| High | 9 | 12.75 (6.00 - 19.51) |  | 7.19 (2.77 - 11.62) |  |
| ***SNAI1*** |  |  | |  | |
| Low | 23 | 30.34 (21.71 - 38.97) | 0.074 | 21.28 (13.07 - 29.48) | 0.148 |
| High | 9 | 15.50 (6.26 - 24.74) |  | 9.44 (1.82 - 17.06) |  |
| ***BCL11A*** |  |  | |  | |
| Low | 23 | 23.50 (15.35 - 31.66) | 0.500 | 17.63 (10.14 - 25.13) | 0.953 |
| High | 9 | 30.39 (17.03 - 43.74) |  | 19.53 (5.88 - 33.18) |  |
| ***AR*** |  |  | |  | |
| Low | 23 | 29.73 (21.53 - 37.92) | 0.062 | 21.50 (13.34 - 29.65) | 0.085 |
| High | 9 | 14.60 (6.32 - 22.88) |  | 9.23 (1.45 - 17.01) |  |
| ***TIMP1*** |  |  | |  | |
| Low | 23 | 32.69 (24.38 - 40.99) | **<0.001** | 23.43 (15.17 - 31.68) | **0.005** |
| High | 9 | 10.00 (3.89 - 16.12) |  | 3.81 (2.40 - 5.22) |  |
| ***CRIPTO1*** |  |  | |  | |
| Low | 23 | 28.19 (19.88 - 36.49) | 0.273 | 19.53 (11.40 - 27.65) | 0.607 |
| High | 9 | 16.12 (8.60 - 23.64) |  | 10.02 (4.19 - 15.85) |  |
| ***GAPDH*** |  |  | |  | |
| Low | 23 | 30.04 (21.23 - 38.84) | **0.046** | 21.79 (13.16 - 30.42) | **0.046** |
| High | 9 | 12.85 (6.68 - 19.02) |  | 6.34 (2.73 - 9.95) |  |

Table S5. Prognosis value of markers identified on CTCs from TNBC patients.

Cut-off value to determine high and low expression was calculated based on percentile 70; CI, confidence interval. P-values were calculated with Log-Rank test.
