# Supplementary figures and images for: Looking for a Better Characterization of Triple-Negative Breast Cancer by Means of Circulating Tumor Cells
Source: J Clin Med. 2020 Jan 27;9(2):353. doi: 10.3390/jcm9020353 (PMC7074553; doi:10.3390/jcm9020353)

**A**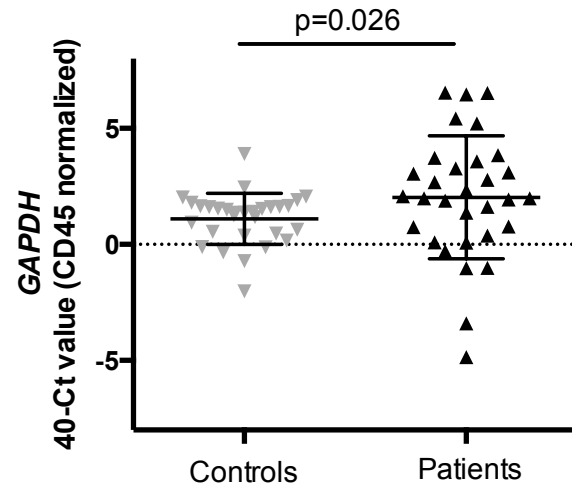**B**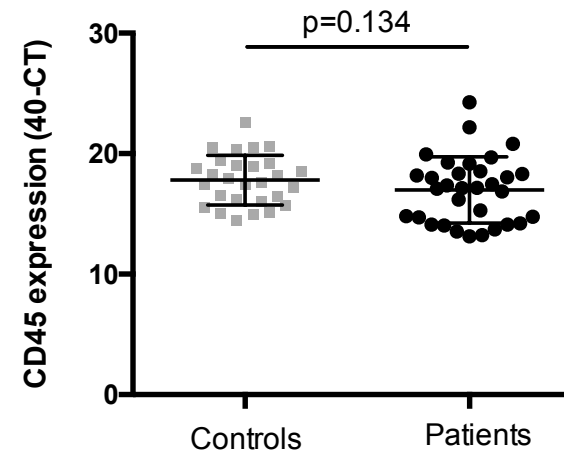**C**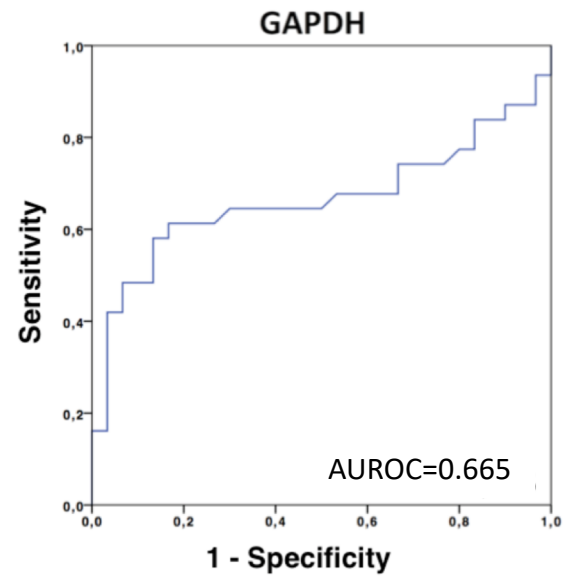**D**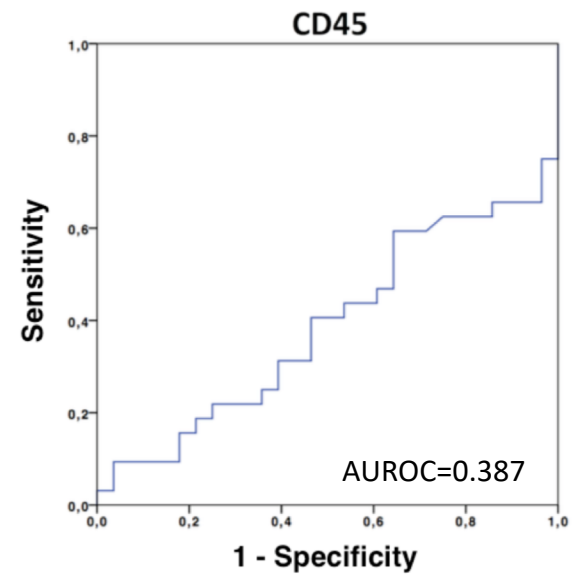

Supplement: Supplementary file 1 [file jcm-09-00353-s001.zip › supp/Figure S1.pdf]

A

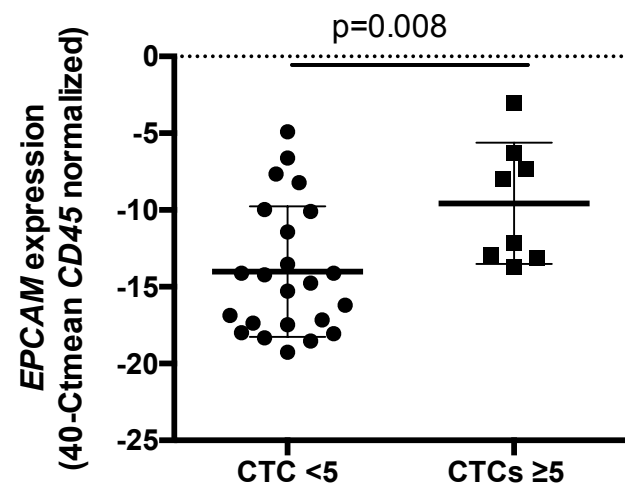

B

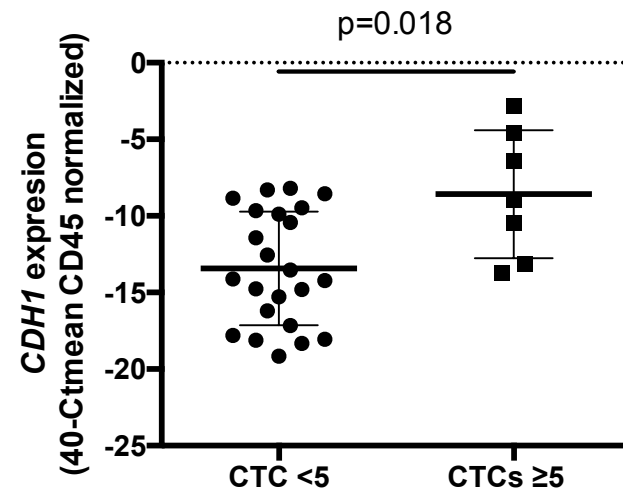

C

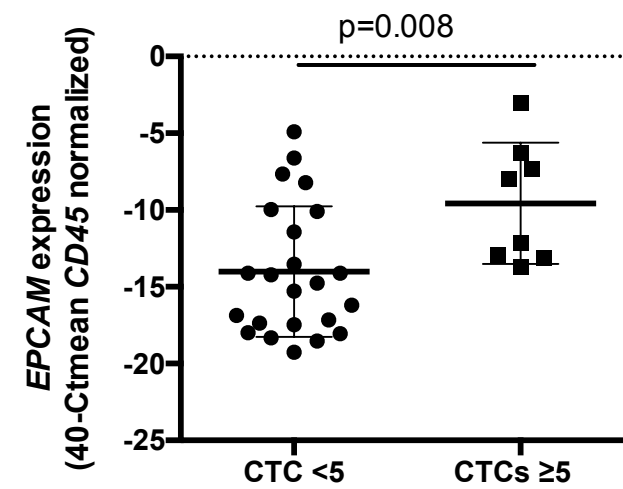

Supplement: Supplementary file 1 [file jcm-09-00353-s001.zip › supp/Figure S2.pdf]

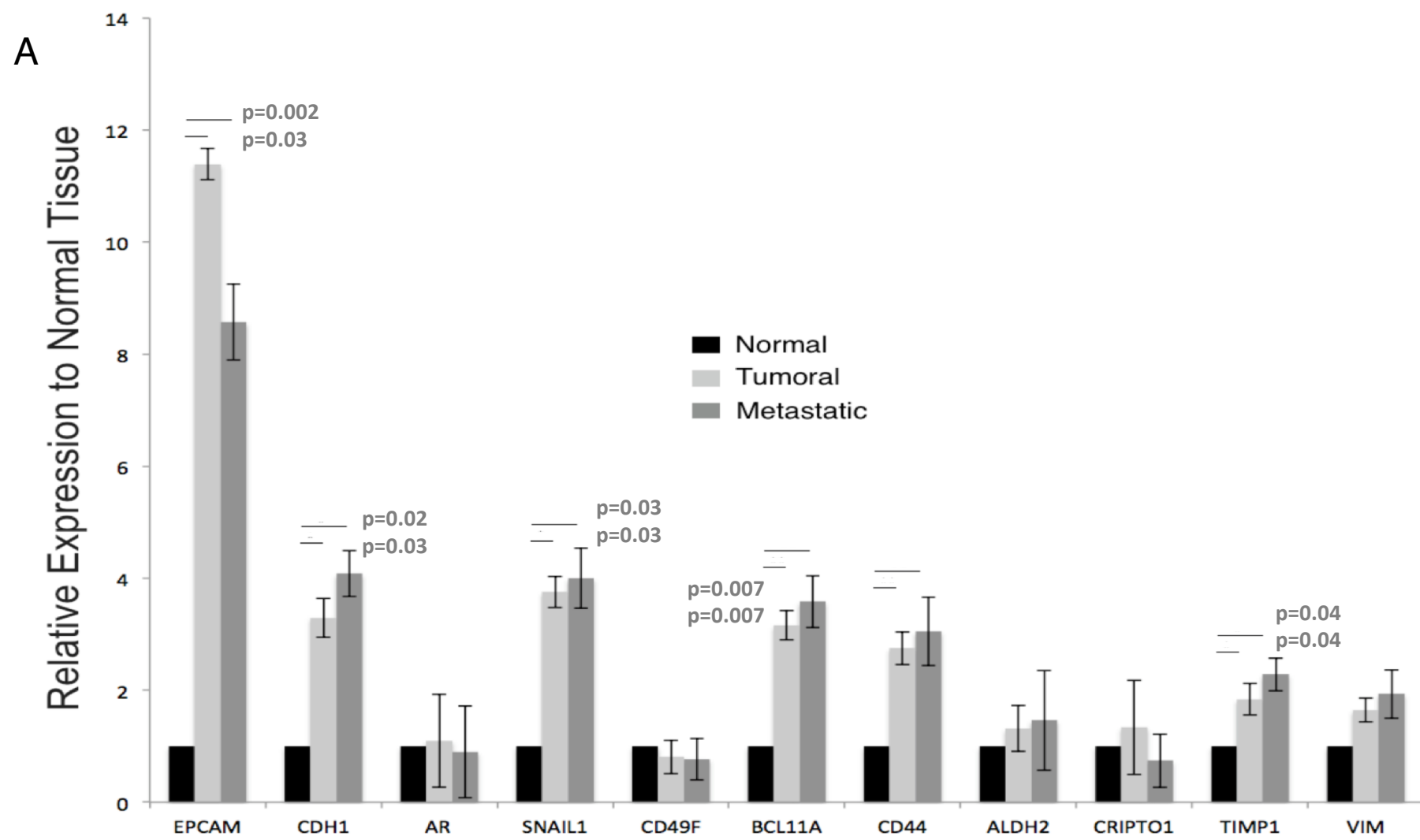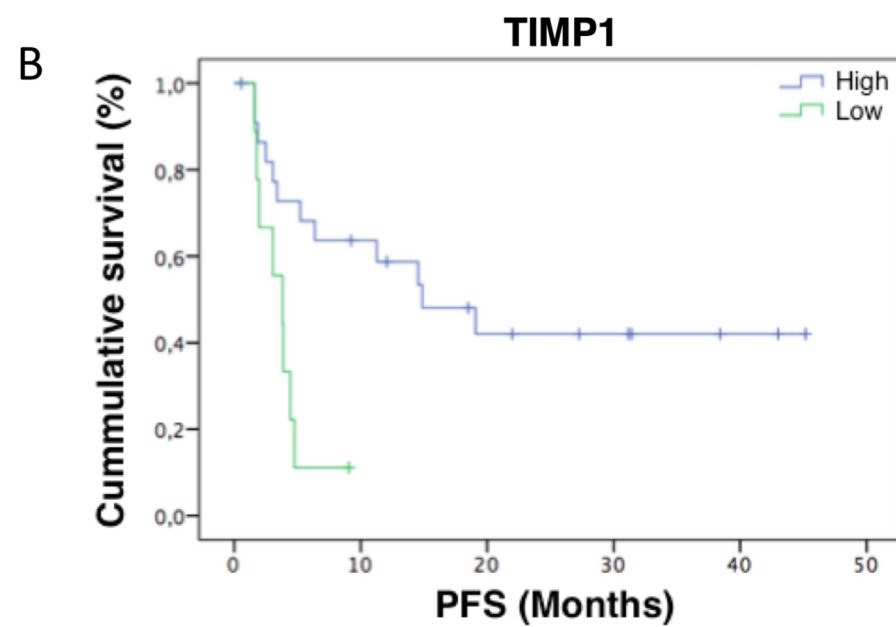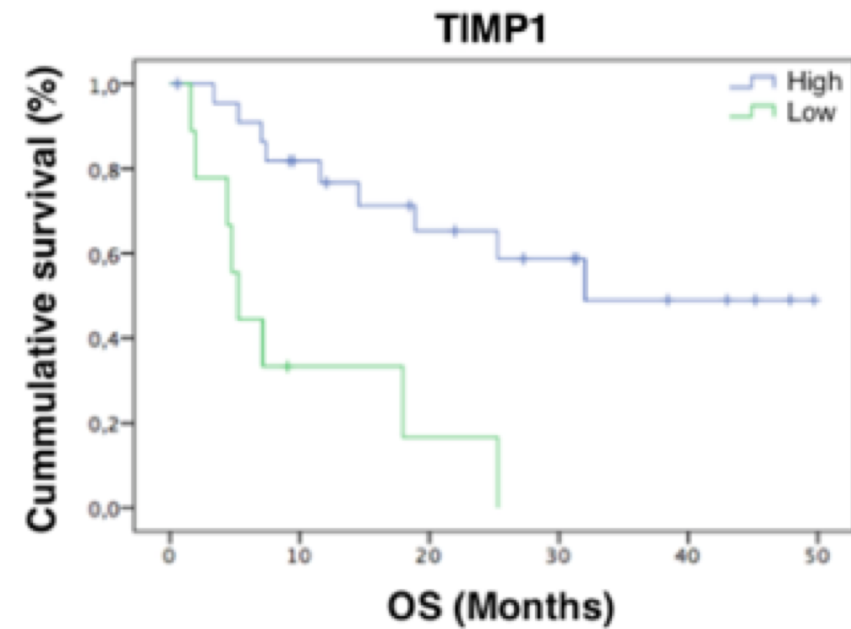

Supplement: Supplementary file 1 [file jcm-09-00353-s001.zip › supp/Figure S3..pdf]

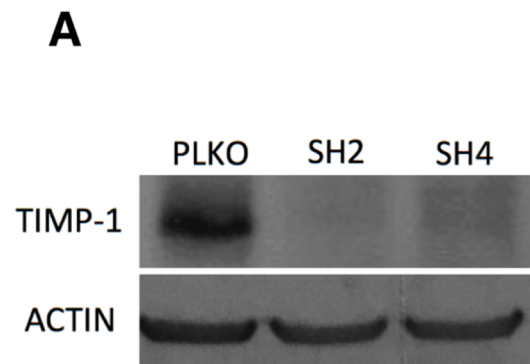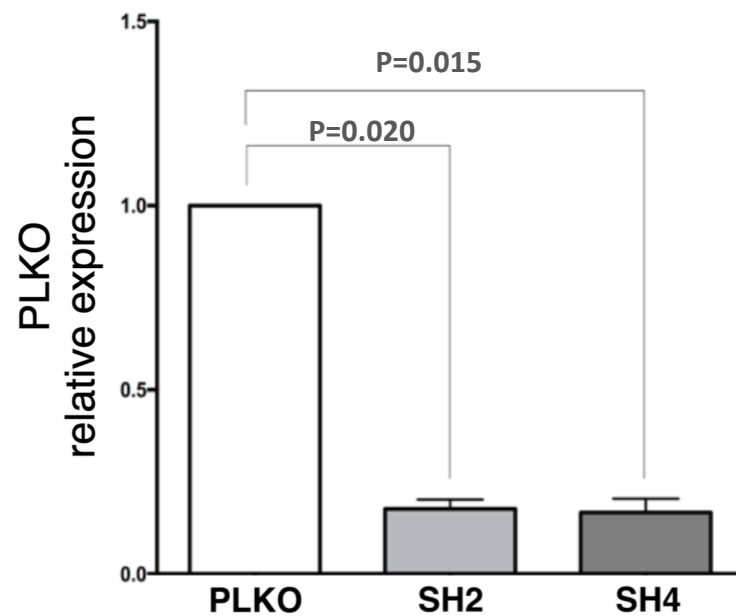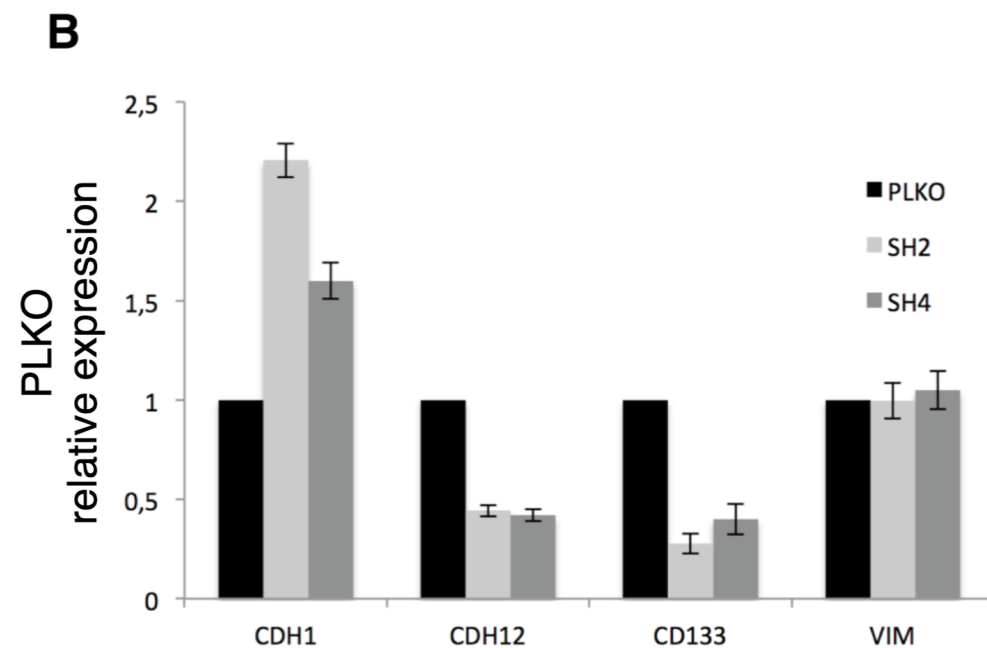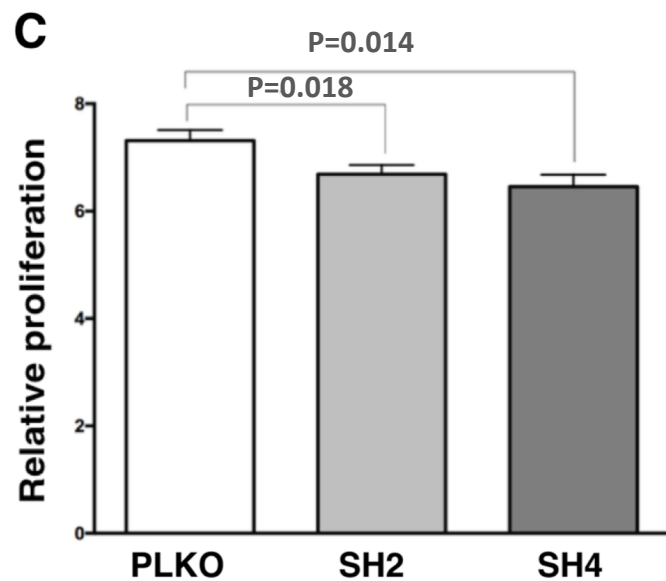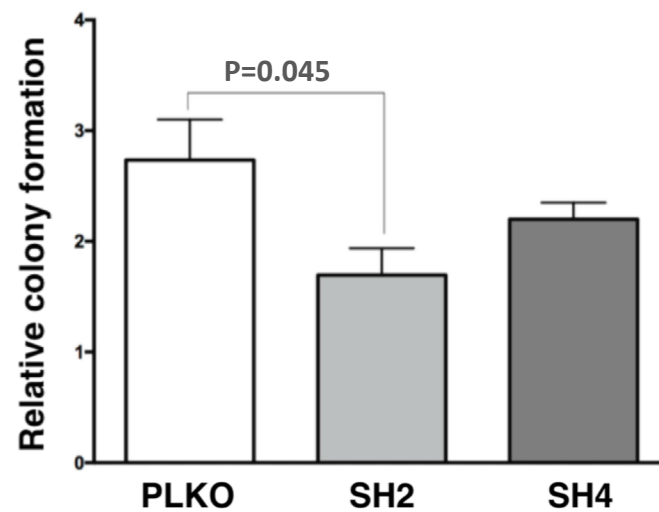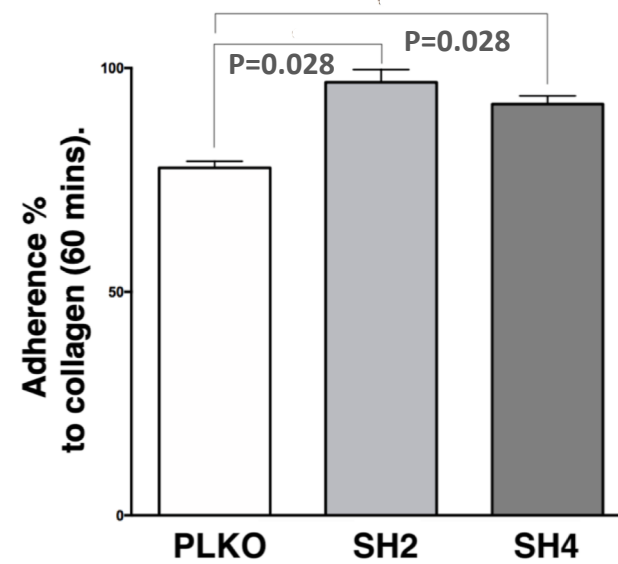

Supplement: Supplementary file 1 [file jcm-09-00353-s001.zip › supp/Figure S4.pdf]

**A**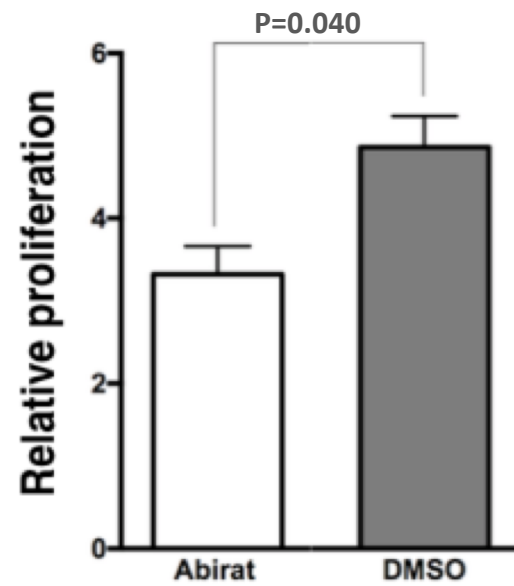**B**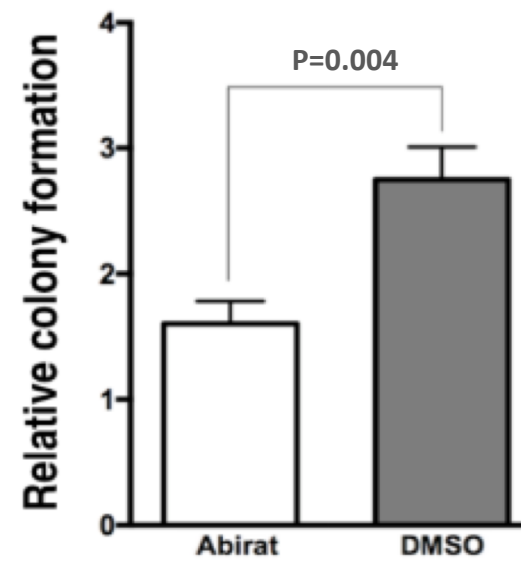**C**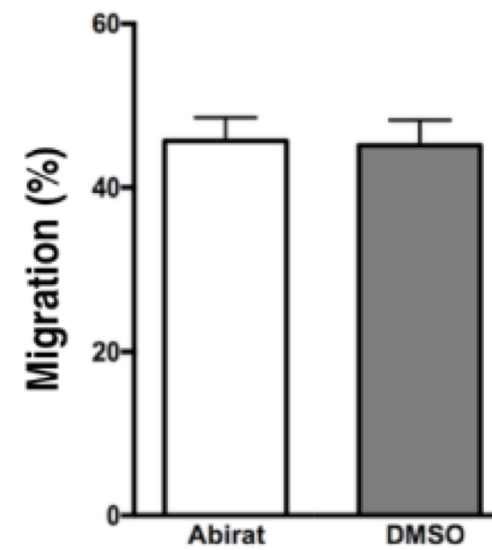

Supplement: Supplementary file 1 [file jcm-09-00353-s001.zip › supp/Figure S5..pdf]

Supplementary Figure 6

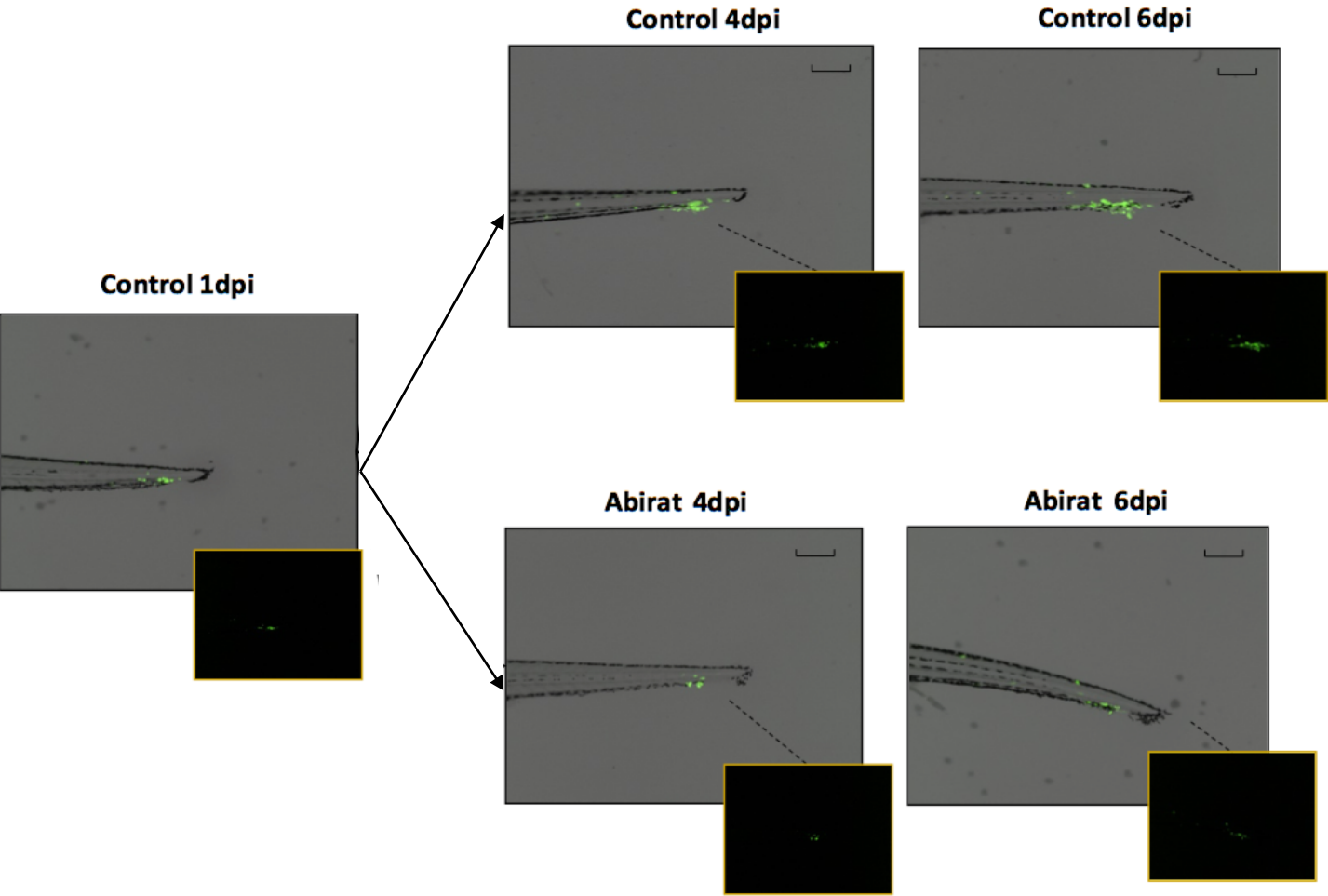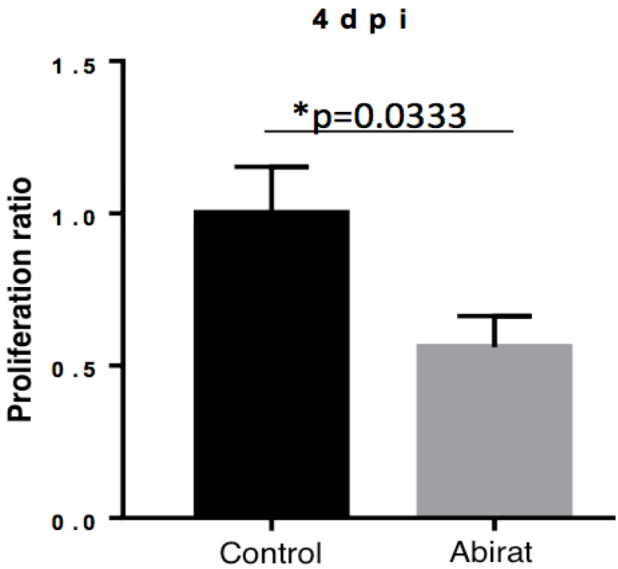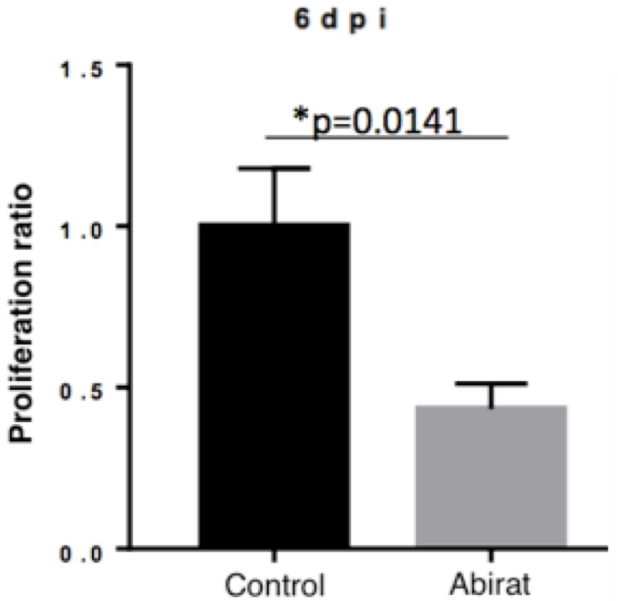

Supplement: Supplementary file 1 [file jcm-09-00353-s001.zip › supp/Figure S6.pdf]
